# Potential Alzheimer’s early biomarkers in a transgenic rat model and benefits of diazoxide/dibenzoylmethane co-treatment on spatial memory and AD-pathology

**DOI:** 10.1038/s41598-024-54156-z

**Published:** 2024-02-14

**Authors:** Charles H. Wallace, Giovanni Oliveros, Lei Xie, Peter Serrano, Patricia Rockwell, Maria Figueiredo-Pereira

**Affiliations:** 1 Department of Biological Sciences, Hunter College CUNY and Graduate Center, 695 Park Ave., New York, NY USA; 2 Department of Computer Sciences, Hunter College CUNY, New York, NY USA; 3 Department of Psychology, Hunter College CUNY, New York, NY USA

**Keywords:** Polypharmacology, Drug repurposing, Alzheimer’s, Potassium channel activator, eIF2α activator, EGR2, HIST1H2AA, Drug discovery, Neuroscience, Diseases

## Abstract

Alzheimer’s disease (AD) is the major form of dementia prevalent in older adults and with a high incidence in females. Identification of early biomarkers is essential for preventive intervention to delay its progression. Furthermore, due to its multifactorial nature, a multi-target approach could be therapeutically beneficial. Our studies included 4- (pre-pathology) and 11-month (mild-pathology) TgF344-AD rats, a transgenic Alzheimer’s model that exhibits age-dependent AD progression. We identified two potential early biomarker genes for AD, early growth response 2 (*EGR2*) and histone 1H2AA (*HIST1H2AA*), in the hippocampus of 4-month females. Out of 17,168 genes analyzed by RNA sequencing, expression of these two genes was significantly altered in 4-month TgF344-AD rats compared to wild-type littermates. We also evaluated co-treatment with diazoxide (DZ), a potassium channel activator, and dibenzoylmethane (DIB), which inhibits eIF2α-P activity, on TgF344-AD and wild-type rats. DZ/DIB-treatment mitigated spatial memory deficits and buildup of hippocampal Aβ plaques and tau PHF in 11-month TgF344-AD rats but had no effect on wild-type littermates. To our knowledge, this preclinical study is the first to report *EGR2* and *HIST1H2AA* as potential AD biomarkers in females, and the benefits of DZ/DIB-treatment in AD. Evaluations across multiple AD-related models is warranted to corroborate our findings.

## Introduction

Alzheimer’s Disease (AD) is the most common form of dementia^1^ and is a multifaceted neurodegenerative disease with aging being the major risk factor^2,3^. Mono-target therapeutics directed at amyloid plaques or tau tangles are common to treat AD, but have a high degree of failure^4^. The use of multi-target drugs, often referred to as polypharmacology, is on the rise^5,6^. In this context and through computational studies^7^, we predicted that diazoxide (DZ) and dibenzoylmethane (DIB) had potential to be repurposed for AD^7^. In fact, DZ or DIB used separately prevented the progression of neurogeneration in preclinical rodent models^8,9^.

DZ has been used for decades for its cardioprotective effects^10^. DZ is a benzothiadiazine derivative that is an agonist to mitochondrial ATP sensitive potassium channels. It increases intracellular K^+^ levels and reduces abnormally elevated Ca^2+^ concentrations commonly found in AD^11^. DZ alone prevented/mitigated cognitive deficits and histopathology in the 3xTgAD mouse model of AD^8^, caspase-dependent apoptosis via BCL-2 activation and BAX inhibition in rat hippocampus^12^, and apoptosis in PC12 cells^13^.

DIB is a β-ketone analog of curcumin found in licorice among other plants^14^. DIB reverses eIF2α-P-mediated translational attenuation occurring in the unfolded protein response, which plays a critical role in controlling protein synthesis rates in cells^9^. This pathway is overactive in brains of AD patients, thus mitigating its activity with DIB could be beneficial^9^. DIB and its derivatives display beneficial effects, such as chemo-preventive, anti-cancer, anti-mutagenic, anti-inflammatory, liver protection, cellular stress preventive, iron-chelating abilities and is even protective against UV rays^9,14–21^. DIB also prevented hippocampal neuronal loss and improved memory deficits in transgenic prion-disease and tauopathy-frontotemporal dementia mouse models^9^. Interestingly, DIB was explored in models of cancer with various combination drug approaches^19,20^.

We investigated the therapeutic potential of chronic treatment with a DZ/DIB combination on the transgenic TgF344-AD (TG-AD) rat model of AD^22^. TG-AD rats develop a wide array of AD pathologies, including cognitive deficits, Aβ plaques, neurofibrillary tangles (NFTs), neuroinflammation and neuronal loss, in an age-dependent progressive manner^22^.

Overall, our studies demonstrate that: (1) at 4 months of age (pre-pathology stage), the expression levels of the *EGR2* and histone *1H2AA* genes were altered in female TG-AD rats, suggesting that they could be early biomarkers for women with AD. (2) the DZ/DIB-treatment mitigates spatial memory deficits and hippocampal Aβ-plaque and neurofibrillary tau-tangle burden in Tg-treated compared to Tg-untreated rats. Thus, our results strongly support that the DZ/DIB-treatment is a potential strategy to mitigate AD pathology due to its multi-target approach.

## Material and methods

### TgF-344 AD rats

Fisher transgenic 344-AD (TG-AD) rats expressing human Swedish amyloid precursor protein (APPsw) and ∆ exon 9 presenilin-1 (PS1-∆E9) both driven by the prion promoter^22^, were purchased from the Rat Resource and Research Center (Columbia, MO). TG-AD (n = 55, Supplemental Table 1) and WT (n = 50, Supplemental Table 1) rats of both sexes were housed in pairs and maintained on a 12-h light/dark cycle with food and water available ad libitum. All animal procedures were performed in compliance with the relevant guidelines and regulations of the Institutional Animal Care and Use Committee (IACUC) at Hunter College. All experimental procedures were approved by the IACUC and were in agreement with the standards outlined in the ARRIVE guidelines.

### DZ/DIB-treatment

**Figure 1:**
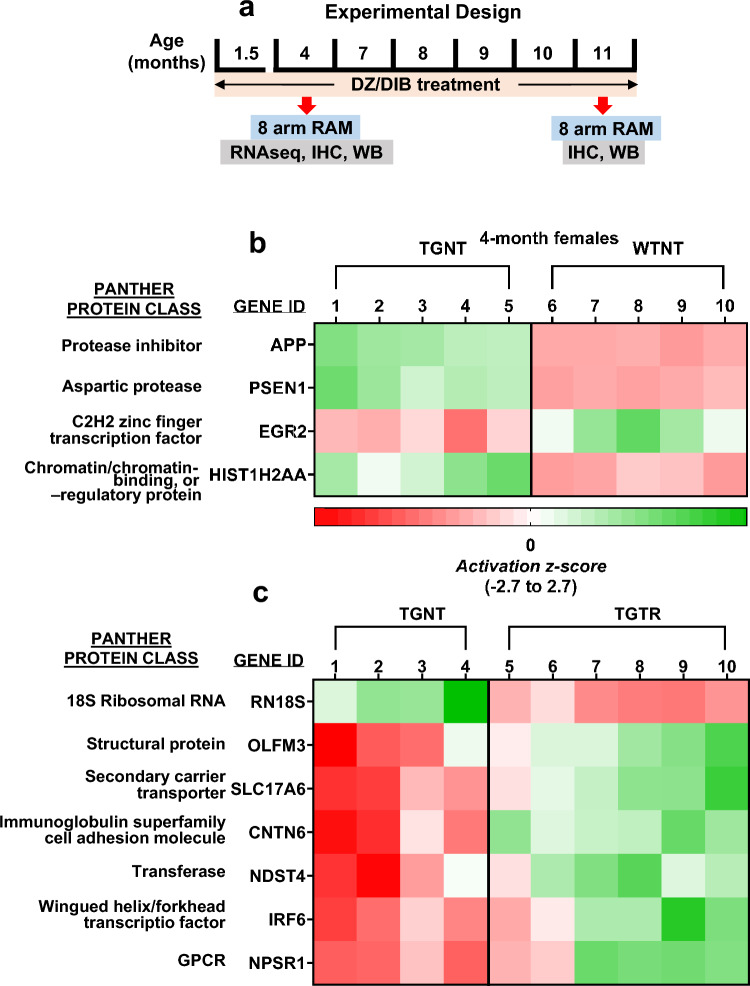
RNAseq heatmaps showing transcriptional changes in WT and TG-AD rats. (**A**) Experimental timeline. (**B**,**C**) Changes in gene expression at 4-months of age in WTNT vs TGNT (**B**) and TGNT vs TGTR (**C**) female rats. Expression levels are represented as changes in activation z-score (ranging from − 2.7 to 2.7). (**B**) Genes *APPsw, PS1-∆E9,* and *HIST1H2AA* are upregulated, while EGR2 is downregulated in 4-month TGNT vs WTNT females. Treatment with DZ/DIB shows downregulation in RN18S, while showing upregulation of OLFM3, SLC17A6, CNTN6, NDST4, IRF6, and NPSR1. Changes in expression are represented as changes in activation z-score (ranging from − 2.7 to 2.7). WTNT, wild-type not treated, TGNT, transgenic-AD not treated, TGTR, transgenic-AD DZ/DIB treated, 8 arm RAM, 8 arm radial arm maze; RNAseq, RNA sequencing; IHC, immunohistochemistry; WB, western blot. (**B**) WTNT: GSM7852852 (WT1), GSM7852853 (WT2), GSM7852854 (WT3), GSM7852855 (WT4), GSM7852856 (WT5). TGNT: GSM7852857 (Tg1), GSM7852858 (Tg2), GSM7852859 (Tg3), GSM7852860 (Tg4), GSM7852861 (Tg5). (**C**) TGNT: GSM7852862 (Tg-1), GSM7852863 (Tg-2), GSM7852864 (Tg-3), GSM7852865 (Tg-4). TGTR: GSM7852866 (Tg-C1), GSM7852867 (Tg-C2), GSM7852868 (Tg-C3), GSM7852869 (Tg-C4), GSM7852870 (Tg-C5), GSM7852871 (Tg-C6).

Orally administered DZ/DIB-treatment started when the rats were 52 days old **(**Fig. 1A). TG-AD and WT rats were fed a combination of DZ (10 mg/kg bw/day, Sigma Aldrich cat# D9035) and DIB (200 mg/kg bw/day, Sigma Aldrich Cat # D33454) in rodent chow (Research Diets Inc. NJ). Non-treated TG-AD and WT rats were fed normal chow. Treatment ended at 4- or 11-months of age when brains were collected for further analyses.

### RNAseq analysis

Right hippocampi were analyzed for gene expression by RNA sequencing (RNAseq) at the UCLA Technology Center for Genomics and Bioinformatics (Los Angeles, CA). Data were normalized as reads per million (RPM) using the TMM method. Differentially expressed genes from DZ/DIB-treated transgenic rats were determined using the edgeR program^23^. RPMs were analyzed for fold-change (FC), p-values, and false discovery rate (FDR) for each gene (Supplemental Table 2). A total of 20 samples originating from 4-month females were analyzed by RNAseq distributed as shown in Fig. 1B,C. Females were chosen because two out of three AD patients are females^24^.

### Radial arm maze (RAM, 8-arm)

**Figure 2:**
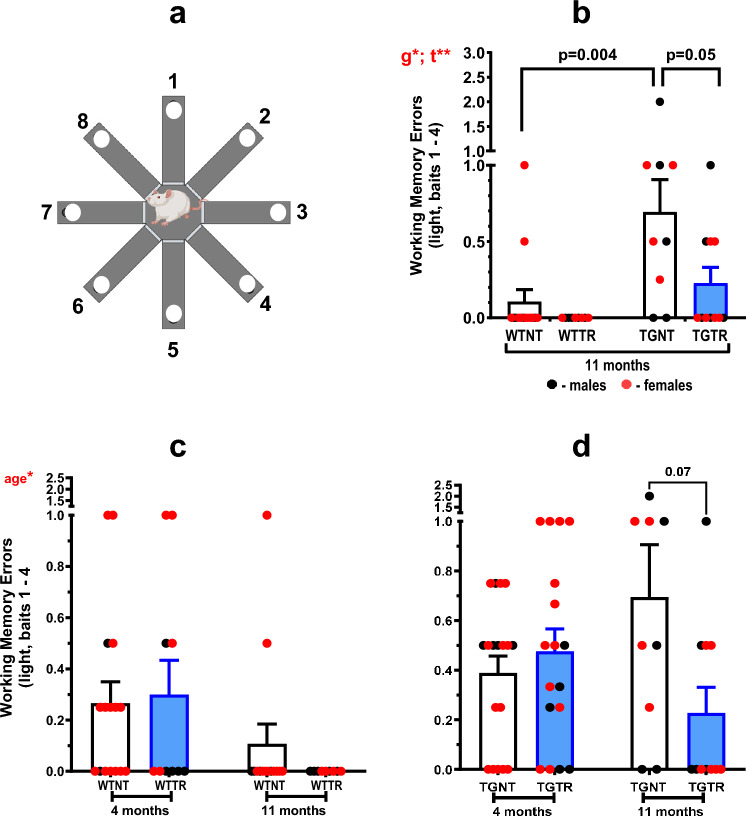
Effects of DZ/DIB-treatment on cognition. (**A**) The 8-arm radial arm maze. (**B**) 11-month old TGNT rats show increased working memory errors under a light working memory load (tasked with reaching the first four arms) when compared to WTNT. DZ/DIB-treatment shows significant reduction in working memory errors in TGTR. (**C**) Age comparisons among wild-type rats show that 11-month wild-type rats exhibit improved working memory compared to 4-month wild-type rats. (**D**) Age-comparison among TG-AD rats do not show any significant working memory differences between 4 and 11-month rats. Repeated measures two-way ANOVA with Sidak’s post-hoc tests were used. *a = age effect, *g = genotype effect, *t = drug treatment effect. * = *p* < 0.05, ** = *p* < 0.01. WTNT, wild-type not treated, TGNT, transgenic-AD not treated, WTTR, wild-type DZ/DIB treated, TGTR, transgenic-AD DZ/DIB treated.

Procedures were as previously described^25^. Briefly, rats received four training trials, two trials per day (Fig. 2A). To ensure the rats were using a spatial strategy, the maze was rotated 90 degrees each day. The sequence of arm entries for each rat to retrieve all the food rewards was recorded. Re-entry into a previously entered arm was scored as an error. We measured the number of errors for light working memory load, defined as the number of errors made collecting baits 1–4, and heavy working memory load, defined as the number of errors in collecting baits 5–8. Rats were food-restricted to 90% of their free-feed weight. Data from a rat was only included if they traveled to all eight arms within 10 min. The number of rats (*n*) for each condition are as follows: RAM at 4-month, 17 WTNT, 18 TGNT, 10 WTTR, and 17 TGTR; RAM at 11 months, 14 WTNT, 9 TGNT, 9 WTTR, and 11 TGTR rats (Supplemental Table 1). Rats were tested on the RAM task at 4 months, at which point, some were sacrificed for RNA sequencing analysis and histology. All remaining rats repeated RAM at 11 months, then sacrificed for biochemical analysis.

### Immunohistochemistry

Hippocampal immunohistochemistry (IHC) was performed in 4- and 11-month old rats, as previously described^26,27^. Primary and secondary antibodies are listed in Supplemental Table 3.

Ramified, reactive, and amoeboid microglia were analyzed by circularity based on the ImageJ form factor as previously described^26,27^.

Sections were imaged with a Zeiss AxioImager M2 wide-field fluorescence microscope combined with a Zeiss AxioCam MRm Rev. 3 camera connected to a motorized stage. The AxioVision 4 software, module MosaicX was used to capture the images saved as ZVI files, loaded onto Image J (NIH, Bethesda, MD), and converted to .tif files for optical density analyses^28^.

### Western blot

Western blots of right hippocampal tissue were generated and quantified as previously described^27^. Different loading controls were used for APP (actin) and eIF2α (β-tubulin) detection due to their respective molecular masses. The same rat samples were used for both westerns and probing antibodies are listed in Supplemental Table 3.

### Statistical analysis

Sexes were combined into four groups: WT and transgenic not treated (WTNT and TGNT) and WT and transgenic DZ/DIB treated (WTTR and TGTR) (Supplemental Table 1, number of rats/group).

Four-way and five-way ANOVAs were used for analysis of NeuN and microglia respectively, and were controlled by Sidak’s multiple comparisons *t*-tests. Three-way ANOVAs were controlled by Tukey’s multiple comparisons *t*-tests, and two-way ANOVAs were used for all other analyses. were controlled by Sidak’s-corrected *t*-tests. All post-hoc analyses used two-tail independent tests. The rolling ball algorithm was used for normalizing pixel intensit^29^. Macroscripts for image processing and quantification were added to GitHub (https://github.com/GiovanniOliveros33/Ibudilast-Manuscript). The alpha level was set at *p* < 0.05 with a 95% confidence interval for each effect. Gene expression analyses involved multiple unpaired *t*-tests with Welch’s correction. For Fig. 1B,C, significant differences were established with a 1.5 fold-change, a *p* < 0.05 and FDR of < 0.05% determined by the two-stage step-up method^30^. Z-scores were generated for RNAseq heatmaps and were calculated with the following formula [(*Sample RPM value*) – (*Mean of all RPM values for respective gene*) / (*Standard Deviation of all RPM values for respective gene*)]. GraphPad Prism version 9.5.1 (La Jolla, California) was used for all statistical analyses in two and three-way ANOVAs, while IBM’s SPSS software was used for four and five-way ANOVA analyses.

## Results

### Changes in EGR2 and HIST1H2AA gene expression as potential early biomarkers for 4-month AD in females; DZ/DIB-treatment up-regulates genes down-regulated in AD and ageing

Approximately two thirds of all Alzheimer's patients are women. Moreover, there is a critical need to identify early biomarkers, for their ability to indicate early stages of AD when treatment can be most effective. Therefore, we compared the transcriptomes of 4-month untreated WT and TG-AD female rats, and the latter with TGTR female rats. The 4-month timepoint was chosen for RNAseq analysis of the expression of 17,168 genes, to reveal changes in pre- or early-AD pathology in females for potential biomarkers. There were four to six rats in each group.

From the 17,168 genes, the expression of only four was significantly different between 4-month WTNT and TGNT female rats. Up-regulation of the APPsw (1.99 fold) and PS1-∆E9 (2.11 fold) genes in TGNT vs WTNT female rats was expected, because besides the endogenous genes, the TG-AD rats express human APPswe and PS1ΔE9 mutations driven by the prion promoter (Fig. 1B). Up-regulation of these two genes served as controls for all conditions as shown in Supplemental Table 2A.

Interestingly, the two other genes were Early Growth Response 2 (*EGR2)* and Histone H2A type 1-A (*HIST1H2AA*) (Fig. 1B, Supplemental Table 2A). *EGR2* exhibited a ~ 2.0 fold decrease in mRNA levels in TGNT vs WTNT females (*p* = 3.5E−06; FDR = 0.015). In contrast, *HIST1H2AA* gene expression was increased by ~ 3.9 fold in TGNT compared to WTNT females (*p* = 1.83E−07; FDR = 0.001). These data suggest that *EGR2* and *HIST1H2AA* maybe early biomarkers for AD in females.

DZ/DIB-treatment increased the expression of six genes: OLFM3 (~ 1.6), CNTN6 (~ 1.8), NDST4 (~ 1.9), IRF6 (~ 2.8), SLC17A6 (~ 1.8), and NPSR1 (~ 4.2) in 4-month female TGTR compared to TGNT (Fig. 1C, Supplemental Table 2B). The RN18S gene was down-regulated (~ 1.6) in TGTR female rats (Fig. 1C, Supplemental Table 2B). Some of these genes that will be discussed below are normally downregulated in AD and/or ageing.

### DZ/DIB-treatment mitigates spatial working memory deficits in 11-months TG-AD rats

A 3-way ANOVA analysis (age x genotype x treatment) of RAM light working memory errors shows an overall effect of age by drug treatment [F_(1,96)_ = 5.640, *p* = 0.0195], and genotype [F_(1,96)_ = 14.46, *p* < 0.0001]. Tukey’s multiple comparisons tests show post-hoc differences between 11-month WTNT and TGNT rats (Supplemental Table 10). Figure 2B shows DZ/DIB-treatment significantly improved spatial working memory of 11-month TGNT (n = 9) vs TGTR (n = 11) under a light working memory load [F_(1,39)_ = 12.25, *p* = 0.001, Sidak’s post-hoc* t* = 2.769; *p* = 0.05]. As expected, 11-months TGNT rats performed significantly worse than WTNT (*n* = 14) rats [(F_(1,39)_ = 6.09, *p* = 0.02), Sidak’s post-hoc (*t* = 3.663; *p* = 0.004)].

A 3-way ANOVA analysis of heavy working memory load showed a significant effects of age [F_(1,97)_ = 19.28, *p* < 0.0001] and genotype [F_(1,97)_ = 6.917, *p* = 0.0099]. Tukey’s multiple comparisons tests show a post-hoc age difference in wild-type rats [*p* = 0.0105, Supplemental Table 10).

### WT rats perform worse in spatial working memory at 4-months than at 11-months and DZ/DB-treatment has no effect on 4-month rats

The light working memory assessment showed that 4-month WT rats perform significantly worse than when retested at 11-months, independently of treatment [Fig. 2C, F_(1–45)_ = 6.27, *p* = 0.02 for age, F_(1–45)_ = 0.16, *p* = 0.69 for drug treatment, no post-hoc differences]. In contrast, there were no significant differences between 4-month and 11-month transgenic rats [Fig. 2D, F_(1–51)_ = 0.07, *p* = 0.80 for age; F_(1–51)_ = 2.89, *p* = 0.10 for drug treatment]. Since 11-month old rats received RAM training also at 4-months, the age effect observed in WT rats could reflect improved recall of prior training, not observed in transgenic rats.

Two-way ANOVA analysis also showed a similar trend in heavy working memory load for 4-month versus 11-month [F_(1,54)_ = 16.45,—< 0.001 for WT; F_(1,43)_ = 4.96, *p* = 0.03 for transgenic]. No significant drug treatment differences were observed for WT [F_(1,54)_ = 0.008, *p* = 0.93], or transgenic rats [F_(1,43)_ = 0.02, *p* = 0.88] (data not shown).

### DZ/DIB-treatment reduces Aβ plaque burden in 11-month TG-AD rats

**Figure 3:**
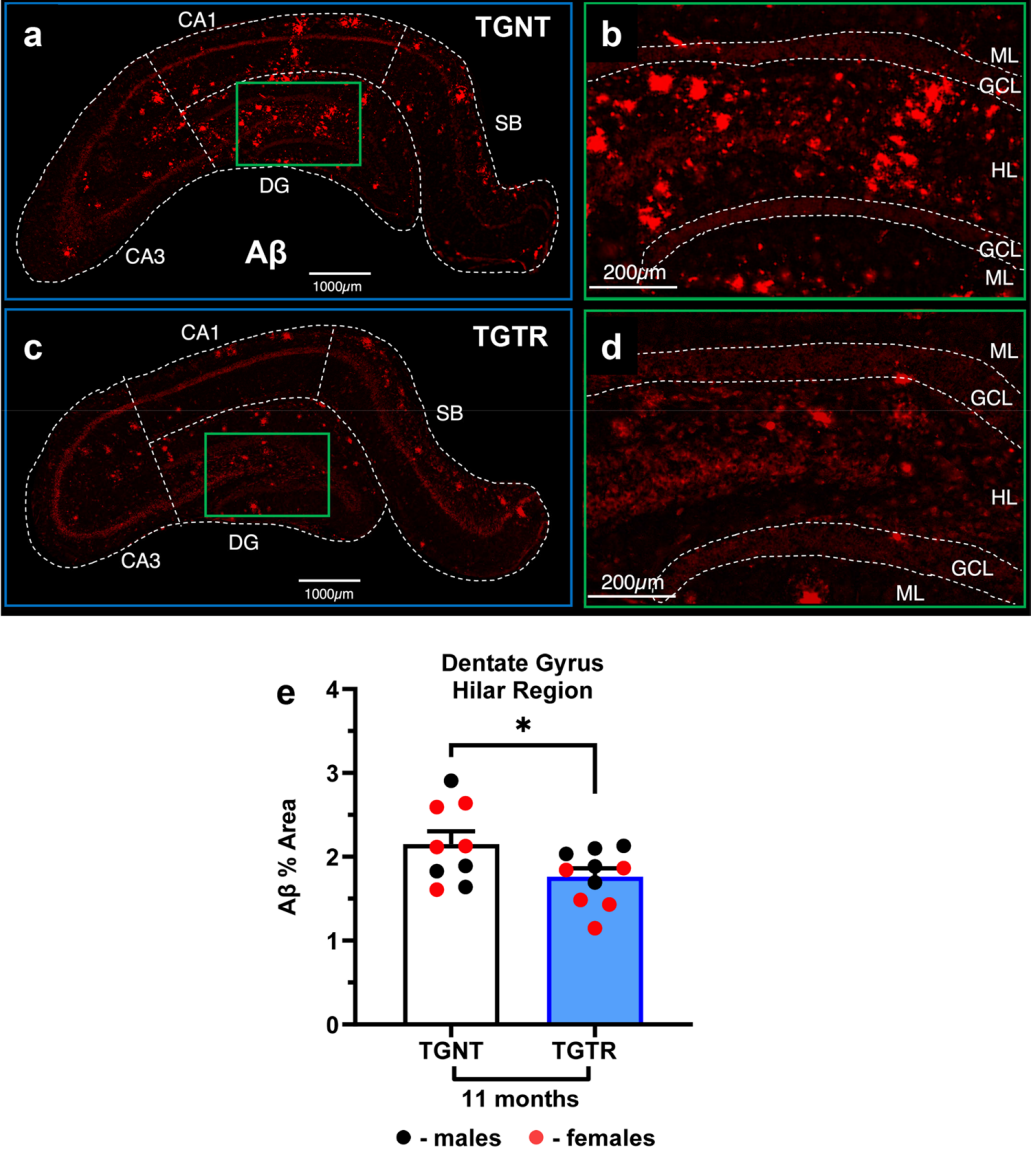
Effects of DZ/DIB-treatment on Aβ plaque burden. 11-month TG-AD rats stained by IHC for amyloid beta plaques across hippocampal regions for TGNT (**A**) and TGTR (**B**) rats. Scale bar = 1000 µm for panels (**A**,**C**), and 200 µm for (**B**,**D**). Dentate Gyrus (DG) magnifications for TGNT (**B**) and TGTR Rats (**D**). Within the dentate gyrus (**B**,**D**), analysis of the hilar region of DG showed DZ/DIB-treatment reduced amyloid beta levels compared to untreated rats (**E**). Unpaired two-tailed *t*-tests with Welch’s corrections were used for analyses performed in panels E. *p < 0.05. TGNT, transgenic-AD not treated; TGTR, transgenic-AD DZ/DIB treated. CA = cornu ammonis, DG = dentate gyrus, SB = subiculum.

Using a two-way ANOVA analysis (hippocampal subregion x drug treatment) shows a significant effect of brain region plaque distribution [F_(5,101)_ = 5.773, *p* < 0.0001]. Sidak’s post-hoc analysis showed region-specific differences for transgenic untreated and DZ/DIB treated rats (Supplemental Table 4). Post-hoc analysis shows DZ/DIB reduced plaque burden in hilar subregion (*p* = 0.0350). DZ/DIB-treatment significantly reduced Aβ plaque burden (red) in the hilar subregion of the left hippocampal DG of 11-month TGTR (Fig. 3C,D) compared to TGNT rats (Fig. 3A,B). A two-tailed unpaired* t*-test with Welch’s corrections performed within each brain region showed reduction of plaques in the hilar subregion (Fig. 3E, Supplemental Table 4: *t* = 2.140, *df* = 17, *p* = 0.05).

DZ/DIB-treatment did not affect Aβ plaque burden throughout the whole left hippocampus and in subregions CA1, CA3, and the SB of 11-month TGTR compared to TGNT rats. All other data are reported in Supplemental Table 4. At 4-months of age TG or WT rats did not develop Aβ plaques independently of drug treatment (Supplemental Fig. 7).

### DZ/DIB-treatment reduces tau PHF1 levels in the hippocampal DG and CA3 of 11-month TG-AD rats

**Figure 4:**
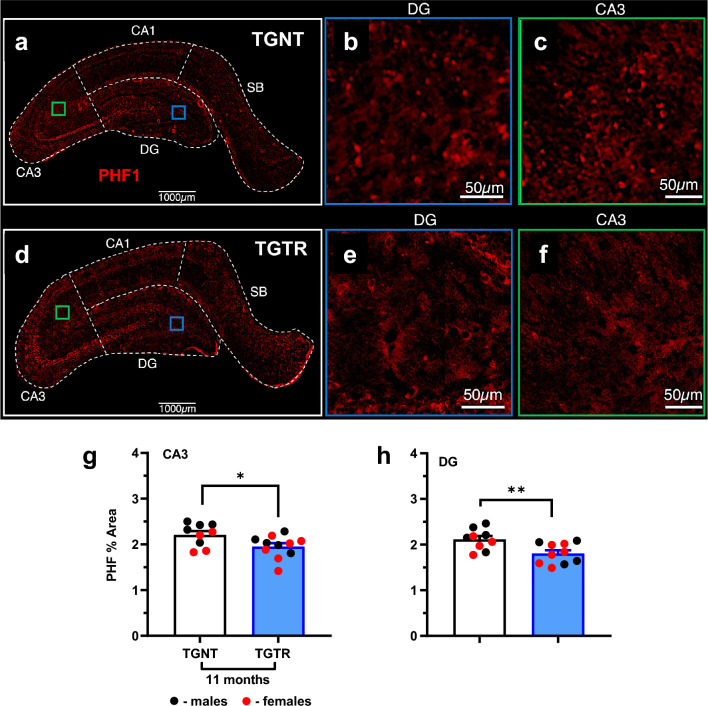
Effects of DZ/DIB-treatment on PHF1 burden. 11 month-old TG-AD rats stained by IHC for Tau paired helical filaments (PHF1), an early precursor of neurofibrillary tangles across hippocampal regions for TGNT (**A**–**C**) and TGTR (**D**–**F**). Scale bar = 1000 µm panels A and D, 50 µm for panels B, C, E, and F. 11 month TGTR rats exhibited significant reductions in PHF1 levels in the dentate gyrus (**E**) and CA3 (**F**). Unpaired two-tailed *t*-tests with Welch’s corrections were used for analyses performed in panels G and H. *p < 0.05, **p < 0.05. TGNT, transgenic-AD not treated; TGTR, transgenic-AD DZ/DIB treated. CA = cornu ammonis, DG = dentate gyrus, SB = subiculum.

DZ/DIB-treatment reduced PHF1 levels in the DG and CA3 hippocampal subregions, as shown by comparing TGNT (Fig. 4A–C) with TGTR (Fig. 4D–F). Two-way ANOVA analysis showed an overall effect of brain region [F_(4,89)_ = 58.73, *p* < 0.0001] with post-hoc differences between TGNT vs TGTR rats (Supplemental Table 5). Region-specific post-hoc analysis shows that DZ/DIB treatment reduced the PHF1 levels in the CA3 (*t* = 2.30, *df* = 17.06, *p* = 0.034, Fig. 4G) and in the DG (*t* = 2.96, *df* = 16.7, *p* = 0.009, Fig. 4H) compared to untreated rats. However, no significant reductions in PHF1 levels were detected in CA1 and SB regions (Supplemental Table 5). We did not analyze tau PHF1 levels in 4-month TG-AD rats since prior reports showed no accumulation of PHF1 until later ages^22^. Staining patterns for PHF1 in wild-type rats, and in the 4-month cohort are shown in Supplemental Fig. 8.

### DZ/DIB-treatment did not alter neuronal loss and microgliosis exhibited by TG-AD rats

Effects of DZ/DIB on neuronal loss were assessed by a 4-way ANOVA, measuring effects of genotype, drug treatment, age, and hippocampal region. 4-way ANOVA showed overall effects of genotype (F_(1, 270)_ = 34.903, *p* < 0.001), treatment (F_(1, 270)_ = 10.713, *p *< 0.001), hippocampal region (F_(4, 270)_ = 20.829, *p* < 0.001), and genotype x age (F_(1, 270)_ 5.351, *p* = 0.022). Post-hoc results are reported in Supplemental Table 6.

Effects of DZ/DIB on microglia levels and morphology were assessed by a 5-way ANOVA, measuring effects of genotype, drug treatment, age, hippocampal region, and morphology. 5-way ANOVA analysis showed overall effects of genotype (F_(1, 1077)_ = 101.936, *p* < 0.001), age (F_(1, 1077)_ = 5.391, *p* = 0.020), region (F_(4, 1077)_ = 12.112, *p* < 0.001), morphology (F_(3, 1077)_ = 7279.614, *p* < 0.001), genotype x age x treatment x region x morphology (F_(15, 1077)_ = 1.870, *p* = 0.023), genotype x age (F_(1, 1077)_ = 67.852, *p* < 0.001), genotype x morphology (F_(3, 1077)_ = 20.836, *p* < 0.001), age x region x morphology (F_(15, 1077)_ = 2.171, *p* = 0.006), and region x morphology y (F_(12,270)_ = 9.689, *p* < 0.001). Overall genotype x treatment trends were also observed (F_(1, 1077)_ = 2.861, *p* = 0.091). Post-hoc results are reported in Supplemental Table 7.

### APP levels are lower in 4- than in 11-month TG-AD rats, and DZ/DB-treatment raises APP levels only in 4-month TG-AD rats

**Figure 5:**
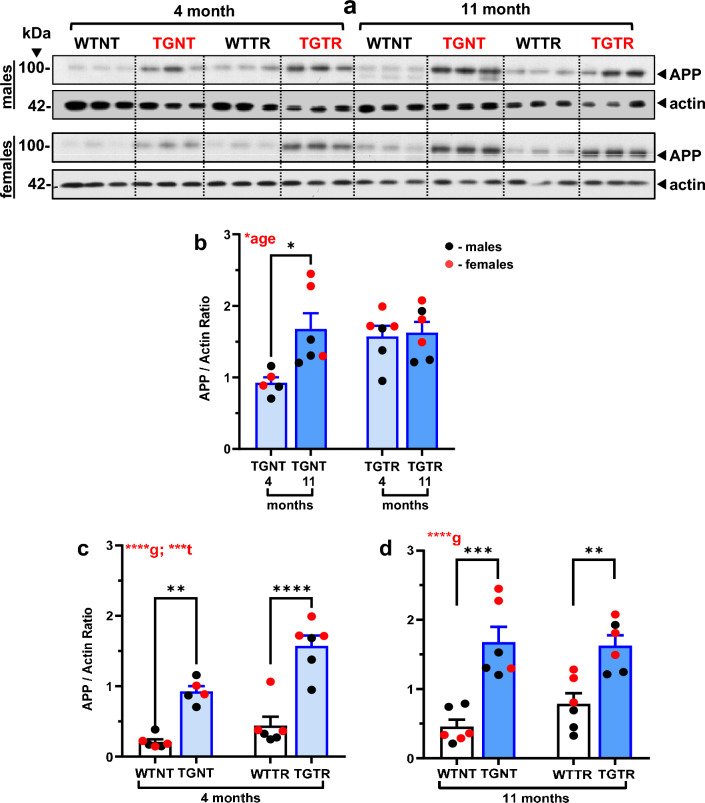
DZ-DIB Treatment effects on APP levels. Male and female rat hippocampal tissue homogenates were used to assess DZ-DIB treatment effects on full-length APP (**A**) levels by western blot analysis. Data represents the percentage of the pixel ratio for full-length APP over actin loading control. Analysis was performed for the four groups WTNT, WTTR, TGNT, TGTR at 4 months and 11 months of age. Graphed data represent the ratio of APP over actin. Values are means + SEM. Significance (asterisks shown on graphs) represent post-hoc effects following an ordinary two-way ANOVA with Sidak’s post-hoc tests. *p < 0.05, **p < 0.01, ***p < 0.001, ****p < 0.0001. WTNT, wild-type not treated, TGNT, transgenic-AD not treated, WTTR, wild-type DZ/DIB treated, TGTR, transgenic-AD DZ/DIB treated; g = genotype effect, t = DZ/DIB drug treatment effect.

Full-length APP levels were assessed by western blotting with the mouse monoclonal antibody 22C11 (Millipore/Sigma), which detects both human and rat APP (Fig. 5A). A three-way ANOVA (age x genotype x treatment) of APP levels shows that TG-AD rats express higher levels of APP compared to age-matched WT rats [*F*_(1, 39)_ = 98.05; *p* < 0.0001] (not shown). Across age, non-treated 11-month TG-AD rats express higher APP levels than 4-month TG-AD rats [*F*_(1, 39)_ = 12.49; *p* = 0.0011]. In addition, DZ/DIB treatment increases APP levels compared to untreated rats, irrespective of genotype [F_(1,39)_ = 8.63, *p* = 0.006]. Significant age and genotype differences were observed (Supplemental Table 8).

Interestingly, a two-way ANOVA analysis shows that APP levels in TGNT rats are higher at 11-months than at 4-months of age [Fig. 5B, F_(1,19)_ = 6.054; *p* = 0.023, with Sidak’s post-hoc differences observed between 4 and 11 months in TGNT rats (*t* = 3.171, *p* = 0.029). This difference could explain why 4-month TGNT rats do not accumulate Aβ plaques. Surprisingly, APP levels are similar in 4-month and 11-month TGTR rats, suggesting that DZ/DIB-treatment increased APP levels only in 4-month TG-AD rats (Fig. 5B, F_(1,19)_ = 3.335; *p* = 0.084].

Two-way ANOVA analysis also showed APP levels were higher in TG-AD than WT rats independently of age and treatment [4-months Fig. 5C; F_(1,19)_ = 71.91; *p* < 0.0001 for genotype; 11 months Fig. 5D, F_(1,20)_ = 40.38; *p* < 0.0001 for genotype. Sidak’s post-hoc differences are shown at 4-months between WTNT and TGNT (*t* = 4.54, *p* = 0.0012), TGNT and TGTR (*t* = 4.108, *p* = 0.0036), and between treated WT and TG rats (*t* = 7.528, *p* < 0.0001), Fig. 5C. Post-hoc differences shown at 11 months between WTNT and TGNT (*t* = 5.321, *p* = 0.0002), Fig. 5D. An additional drug treatment effect was observed at 4 months [F_(1,19)_ = 16.30; *p* = 0.0007, Fig. 5C]. This is expected, since TGNT rats overexpress human APPswe and endogenous rat APP, while WT rats only express endogenous APP.

### eIF2α levels are higher in TG-AD than WT rats, and DZ/DIB-treatment attenuates eIF2α levels

**Figure 6:**
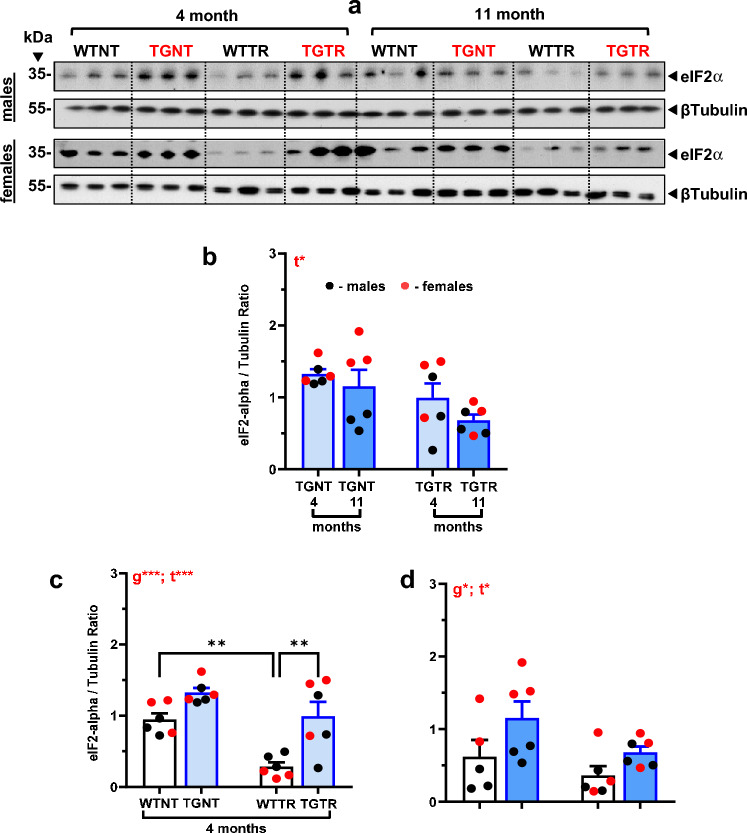
DZ-DIB Treatment effects on eIF2α levels. Male and female rat hippocampal tissue homogenates were used to assess DZ-DIB treatment effects on eIF2α (**A**) levels by western blot analysis. Data represents the percentage of the pixel ratio for eIF2α over β-tubulin loading control. Analysis was performed for the four groups WTNT, WTTR, TGNT, TGTR at 4 months and 11 months of age. Graphed data represent the ratio of eIF2α over β-tubulin. Values are means + SEM. Significance (asterisks shown on graphs) represent post-hoc effects following an ordinary two-way ANOVA with Sidak’s post-hoc tests. *p < 0.05, ***p < 0.001. WTNT, wild-type not treated, TGNT, transgenic-AD not treated, WTTR, wild-type DZ/DIB treated, TGTR, transgenic-AD DZ/DIB treated; g = genotype effect, t = DZ/DIB drug treatment effect.

The translational control factor eIF2α along with its phosphorylating kinases such as PERK, acts as a critical molecular switch that inhibits global protein translation during the unfolding protein response (UPR)^9^. This molecular switch is overactive in AD, thus attenuating its activity would be beneficial to AD^9,31^. We assessed the levels of eIF2α by western blotting (Fig. 6A). A three-way ANOVA (drug treatment x age x genotype) revealed an overall attenuating effect of DZ/DIB treatment on eIF2α levels irrespective of age and genotype [F_(1, 39)_ = 17.06; *p* = 0.0002] (not shown). Interestingly, eIF2α levels were increased in TG-AD rats compared to WT littermates, independently of age and treatment [F_(1, 39)_ = 21.45; *p* < 0.0001]. No age specific effects were observed [*F*_(1, 39)_ = 3.09; *p* = 0.086, Supplemental Table 9].

A two-way ANOVA (age x drug treatment) for age-specific effects show DZ/DIB-treatment lowered eIF2α levels in TGTR rats of both ages, compared to TGNT rats [Fig. 6B, F_(1,20)_ = 6.264; *p* = 0.0211], with no post-hoc differences observed. This effect could be associated with DIB’s capacity to inhibit eIF2α activity. eIF2α levels were higher at 4- (Fig. 6C) and 11-months (Fig. 6D) in TGNT rats than in their WTNT littermates [4-months, F_(1, 20)_ = 20.94, *p* = 0.0002; 11-months, F_(1,19)_ = 5.95, *p* = 0.03], assessed by two-way ANOVA. These data are consistent with AD patients exhibiting higher eIF2α activity than controls. We also observed an overall drug effect, where DZ/DIB attenuated eIF2α levels irrespective of genotype [F_(1,20)_ = 17.61, *p* = 0.0004], with Sidak’s post-hoc differences observed between WTTR and WTNT rats (*t* = 3.952, *p* = 0.0047) at 4 months); [F_(1,19)_ = 4.396, *p* = 0.0496] at 11 months, with no post-hoc differences observed.

## Discussion

Our data established that Tg-AD rats manifest cognitive deficits and signs of AD-pathology at 11- but not at 4-months of age. We included 4-month (pre-pathology) Tg-AD and WT rats in our studies, to potentially identify early AD biomarkers.

As expected, RNAseq analysis in 4-month female rats showed significant up-regulation of *APP* and *PSEN1* genes in TGNT vs WTNT rats, because Tg-AD rats express human *APPsw* and *PS1-∆E9* both driven by the prion promoter^22^. Notably, out of 17,168 genes assessed, there are only two other genes the expression of which is significantly different between the two genotypes, i.e. *EGR2* and *HIST1H2AA*. Our RNAseq data showed a ~ twofold decrease in EGR2 expression in 4-month TGNT compared to WTNT female rats. EGR proteins have long been investigated in the CNS for their role in controlling gene expression^32^. EGR2 is an immediate early gene and an inducible transcription factor^33^. EGR2 is also a SUMO E3 ligase for its coregulators, the Nab proteins^34^. The expression of EGR2 is regulated by NF-κB^35^, and its deletion is lethal in mice^36^. Furthermore, EGR2 expression levels were strongly induced in rat^37^ and mouse^35,38^ hippocampi using the LTP paradigm, supporting that EGR2 plays a critical role in LTP stabilization. In contrast, one study demonstrated improved motor skill learning and enhanced 24 h long-term memory in conditional mutant mice lacking Egr2 forebrain expression^39^. Additional studies are required to sort out the role of Egr2 in cognition. There are very few studies on Egr2 and its possible roles in AD due to the perinatal lethality of this deletion in animal models^40^. Our current study with an AD rat model, suggests that Egr2 could be a potential biomarker for AD in females.

*HIST1H2AA* is a subtype of the H2A histone which is one of five histones that package DNA to make up the nucleosome, which is important for gene regulation^41^. Histones regulate gene expression, and they are post-transcriptionally modified with mechanisms such as acetylation, methylation, phosphorylation and ubiquitination^42^. A study using human frontal cortex brain tissue found that H2A was less ubiquitinated in AD brain tissue compared to age matched controls^43^. Therefore, the increase in *HIST1H2AA* could result from a reduction in ubiquitin/proteasome degradation. Further studies are required to conclude if *HIST1H2A* upregulation is a result of less ubiquitin/proteasome-dependent degradation.

The RNAseq analysis in TGTR vs TGNT rats was performed in females at 4 months of age to assess how the DZ/DIB-treatment may alter gene expression in the pre-pathology stage of AD pathogenesis in women. Genes that were upregulated are connected to cognition, neurogenesis, differentiation, synaptic plasticity, apoptosis, and amyloid toxicity. The specific genes that were up regulated in TGTR vs TGNT rats were: (1) Olfactomedin 3 (*OLFM3*), which is important for differentiation in the brain and retina^44^. (2) Solute Carrier Family 17 Member 6 (*SLC17A6*) that enables the activity of glutamate transporters and is important for synaptic plasticity and expression correlated to cognitive function^45,46^. 3) Contactin 6 (*CNTN6*) that plays a role in axonal formation for the mossy fibers within the hippocampus during development, and is also thought to be involved in apoptosis for neuron survival^47^. (4) N-deacetylase and N-sulfotransferase 4 (*NDST4*) identified as a marker for recently differentiated neural cells^48^. (5) Interferon regulatory factor 6 (*IRF6*), which is relevant to epithelial tissue, where it mediates differentiation and helps regulate apoptosis^49,50^. (6) Neuropeptide S receptor 1 (*NPSR1*) is important in hippocampal function and promotes synaptic plasticity^51^. Downregulation of the 18S ribosomal RNA (*RN18S*) house-keeping gene, indicates ribosomal dysfunction leading to decreased protein synthesis, which is known to be an early event in AD^52^.

Interestingly, the *SLC17A6* and *NDST4* genes that are upregulated after DIB/DZ treatment, are known to decline with AD severity and/or ageing^46,48,53^. It is possible that the pathology that contributes to a decline in the transcriptome of the TGNT may have been mitigated by the DZ/DIB-treatment at 4-months of age. The long-term effects of treatment on these proteins during moderate pathology is unknown and should be explored at later disease phenotypes.

As expected, 11-month TG-AD rats performed worse in spatial working memory than their WT littermates. However, WT rats at 4-months performed worse than when they were retested at 11-months. This age effect could reflect improved recall of prior training in the 11-month WT rats, which was not observed in transgenic rats of the same age. The latter already developed mild cognitive deficits and AD-pathology at 11-months.

The cognitive assessment at 11-months of age shows that DZ/DB treatment mitigated spatial deficits in light working memory load performance in TGTR vs TGNT with an overall treatment benefit across all groups indicating an effect beyond genotype. Additionally, there was an age-dependent decline in heavy working memory load irrespective of genotype likely because this stage of the radial arm maze becomes increasingly challenging where age related decline is detected.

The DZ/DIB treatment covered a wide range of targets including common markers for AD as well as specific targets for DZ and DIB. DZ/DIB-treatment showed reductions in Aβ plaque and tau PHF1 loads. This is consistent with prior investigations using the individual drugs^8,9^. Interestingly, APP protein levels in TGNT rats were higher at 11-month than at 4-months, indicating why 4-month rats do not accumulate Aβ plaques. It is important to note that the RNAseq analyses showed that the levels of APP mRNA overexpression is the same in 4-month (Fig. 1B and Supplemental Table 2A) and 11-month (Supplemental Table 2C, data from^27^) TGNT rats when compared to age matched WTNT rats. On the other hand, DZ/DIB-treatment increased APP levels in 4-month but not 11-month TGTR rats. DZ/DIB-treatment could be maintaining protein translation and showing some neuroprotective potential due to a reduction in eIF2α expression in the overall hippocampus. eIF2α is a subunit within the eIF2 initiation complex responsible for regulating global and specific mRNA translation, required for learning and memory as well as for neuronal integrity maintenance in health and disease^54^. Therefore, DZ/DB-treatment reduces the likelihood for premature translational depression, consistent with prior DIB treatment investigations^9^. DZ/DIB may also be working through a mechanism of maintaining synaptic plasticity, which is not reflected by just assessing the levels of mature neurons. Diazoxide is known to amplify AMPA receptor currents in the hippocampus^55^. Moreover, in the TgF344-AD rat model elevated AMPA subunit receptor expression is proposed to improve cognition in females despite increases in plaque burden^56^.

Treatment with these two drugs widens the reach of polypharmacology, with multiple mechanisms of action such as DZ balancing calcium overload, DIB relieving cellular stresses through eIF2α. DZ increases intracellular potassium levels and conversely reduces intracellular calcium levels, while DIB has a host of effects on signaling pathways involved in cancer, mitigating inflammation and cellular stress, and neuroprotection^16^.

Our study identified two potential biomarkers for pre-symptomatic AD in females: *EGR2* and *HIST1H2AA*. In addition, we found that early (at 4-months) DZ/DIB-treatment induced the upregulation of some genes known to be downregulated in AD. Some of these genes are involved in neurogenesis and other protective mechanisms. Overall, we investigated the repurposing potential for DZ and DB as a combination treatment for AD. DZ has been used for decades for its cardioprotective effects, showing it is a well-tolerated and efficacious drug, therefore a great candidate for repurposing^10^. The mechanism of DZ against apoptosis may be helpful in preventing neuronal loss in later stages of AD. DIB is a promiscuous drug with affinity for many targets and likely some still unknown. Some of the suggested mechanisms for DIB are inhibition of the unfolded protein response, increase in activity of protective transcription factors, induction of cell cycle arrest, and reducing expression of androgen receptors^9,21,57^. DIB was shown to be neuroprotective to hippocampal histopathology^9^, but to our knowledge, not in an AD animal model.

Although current FDA approved AD therapeutics are managing symptoms to varying degrees of success, they currently do not address biological changes that initiate the cascade of changes observed in disease pathology and progression. A likely culprit is the multifactorial nature of AD, which multi-drug therapies have the potential to address through targeting of multiple signal pathways and mechanisms that contribute to AD progression. Shifting efforts to computational models and polypharmacology is on the rise and can prove to be an invaluable asset, to which our findings have come to suggest.

### Supplementary Information

Supplementary Information.

## Data Availability

The datasets generated and/or analyzed during the current study are available in the NIH GEO repository: https://www.ncbi.nlm.nih.gov/geo/query/acc.cgi?acc=GSE245979.
