# Supplementary material for: Potential Alzheimer’s early biomarkers in a transgenic rat model and benefits of diazoxide/dibenzoylmethane co-treatment on spatial memory and AD-pathology
Source: Sci Rep. 2024 Feb 14;14:3730. doi: 10.1038/s41598-024-54156-z (PMC10867006; doi:10.1038/s41598-024-54156-z)
Supplement: Supplementary file 1 — Supplementary Information. [file 41598_2024_54156_MOESM1_ESM.pdf]

Potential Alzheimer's early biomarkers in a transgenic rat model and benefits of diazoxide/dibenozylmethane co-treatment on spatial memory and AD-pathology

Charles H. Wallace; Hunter College CUNY (Department of Biological Sciences), New York (New York) USA

Giovanni Oliveros; Hunter College CUNY (Department of Biological Sciences), New York (New York) USA

Xie, Lei; Hunter College CUNY, (Department of Computer Sciences), New York (New York) USA

Serrano, Peter; Hunter College CUNY (Department of Psychology), New York (New York) USA

Rockwell, Patricia; Hunter College CUNY (Department of Biological Sciences), New York (New York) USA

Figueiredo-Pereira, Maria; Hunter College CUNY (Department of Biological Sciences), New York (New York) USA

Supplemental Table 1 – Number of rats used in our study.

	Untreated		DZ (10mg/kg/day) + DIB (200mg/kg/day)	
**Genotype**	**Wild Type**	**Transgenic-AD**	**Wild Type**	**Transgenic-AD**
**Age**	**Four Months**			
**# of Rats**	**17**	**18**	**10**	**17**
**Age**	**11 Months**			
**# of Rats**	**14**	**9**	**9**	**11**
**Total # of Rats (105 Rats)**	**31**	**27**	**19**	**28**

Supplemental Table 2A – RNA sequence analysis for differentially expressed genes in 4-month female TGNT vs. WTNT rats.

Gene symbol	Fold change	*P* value	FDR	TGNT RPM mean ± SEM (*n*=5)	WTNT RPM mean ± SEM (*n*=5)
APP	↑ 1.96	1.82E-37	3.12E-33	2175.8 ± 66.4	1107.8 ± 20.3
PSEN1	↑ 2.09	2.28E-29	1.96E-25	74.1 ± 3.8	35.4 ± 1.1
EGR2	↓ 2.04	3.50E-06	0.015	3.9 ± 0.5	8.0 ± 0.7
HIST1H2AA	↑ 3.92	1.83E-07	0.001	2.0 ± 0.2	0.5 ± 0.1

Supplemental Table 2B – RNA sequence analysis for differentially expressed genes in 4-month female TGTR vs. TGNT rats.

Gene symbol	Fold change	*P* value	FDR	TGTR RPM mean ± SEM (*n*=6)	TGNT RPM mean ± SEM (*n*=4)
RN18S (LOC310926)	↓ 1.60	6.40E-10	1.10E-05	2160.9 ± 79.5	3447.7 ± 286.2
Olfm3	↑ 1.60	1.04E-05	0.029	36.9 ± 2.1	23.0 ± 3.5
Slc17a6	↑ 1.82	1.46E-08	1.04E-04	31.1 ± 2.2	17.1 ± 1.9
Cntn6	↑ 1.82	3.57E-06	0.015	10.9 ± 0.4	6.0 ± 0.9
Ndst4	↑ 1.85	1.42E-05	0.030	32.2 ± 2.2	17.4 ± 3.8
Irf6	↑ 2.78	2.06E-05	0.039	6.5 ± 1	2.3 ± 0.6
Npsr1	↑ 4.15	1.20E-05	0.029	1.8 ± 0.3	0.4 ± 0.2

Supplemental Table 2C – RNA sequence analysis for differentially expressed genes in 11-month female TGNT vs. WTNT rats.

Gene symbol	Fold change	P value	FDR	TGNT RPM mean ± SEM (n=5)	WTNT RPM mean ± SEM (n=5)
APP	↑ 2.09	2.37E-07	0.002038	2897.9 ± 73.1	1383.4 ± 72.3
PSEN1	↑ 2.20	1.83E-14	3.15E-10	66.1 ± 3.1	30.1 ± 1.9
EGR2	↓ 1.30	0.417848	1	4.8 ± 1.1	6.2 ± 1.6
HIST1H2AA	↑ 5.27	0.017537	0.872118	1.6 ± 1.4	0.3 ± 0.1

Supplemental Tables 2A, 2B and 2C - RNA sequence analysis for differentially expressed genes in (A) 4-month female transgenic AD non-treated (TGNT) compared to age-matched female wild-type non-treated (WTNT) rats. **(B)** 4-month female transgenic AD DZ-DIB treated (TGTR) rats compared to age-matched female TGNT rats. **(C)** 11-month female transgenic AD non-treated (TGNT) compared to age-matched female wild-type non-treated (WTNT) rats. Genes are listed with significant fold change difference in expression. Upregulation in gene expression is represented with an up-arrow and downregulation in gene expression represented with a down-arrow. Fold change, *p* values, false discovery rates (FDR), means, and standard errors of the mean (SEM) for RPMs (reads per million). All genes with an FDR under 0.05 were included in A and B, but not in C.

Supplemental Table 3 - Antibodies used for IHC and WB analyses of hippocampal tissue.

Antibody	Company	Catalog Number	Species/Type	Dilution	Assay
PRIMARIES					
**Aβ (4G8)**	Biolegend	#800708	Mouse Monoclonal	1:1000	IHC
**eIF2α**	Cell Signaling	#2103S	Mouse Monoclonal	1:1,000	WB
**FL-APP (22C11)**	Millipore Sigma	#MAB348	Mouse Monoclonal	1:2000	WB
**Iba1**	Synaptic Systems	#234006	Chicken Polyclonal	1:500	IHC
**NeuN**	Millipore Sigma	#ABN91	Chicken Polyclonal	1:500	IHC
**PHF1**	Donated by Dr. Peter Davies	N/A	Mouse Monoclonal	1:80	IHC
**β-Actin (AC-74)**	Sigma-Aldrich	#A2228	Mouse Monoclonal	1:10000	WB
**β-Tubulin (TUBB3)**	Covance	#MMS-435P	Mouse Monoclonal	1:10000	WB
SECONDARIES					
**Alexa Fluor 488, Goat anti-Mouse IgG (H+L)**	ThermoFisher	#A-21202	Mouse Secondary	1:250	IHC
**Alexa Fluor 488, Goat anti-Chicken IgY (H+L)**	ThermoFisher	#A-11039	Chicken Secondary	1:250	IHC
**Alexa Fluor 568, Goat anti-Rabbit IgG (H+L)**	ThermoFisher	#A-11011	Rabbit Secondary	1:250	IHC

Supplemental Table 3 - Abbreviations: IHC, immunohistochemistry; WB, western blot; Aβ, amyloid β; eIF2α, eukaryotic translation initiation factor; FL-APP, full length amyloid precursor protein; Iba1, ionized calcium binding adaptor molecule 1; NeuN, neuronal nuclei; neuronal marker; PHF1, paired helical filaments

Supplemental Table 4: Effects of DZ/DIB on Amyloid Beta (Aβ 4G8 antibody) levels across hippocampal regions at 11 months.

Two-way ANVOA Analysis					
Source of Variation	% of total variation	P value	P value summary	Significant?	P value
Interaction	9.091	0.0305	*	Yes	
Drug Treatment	0.1579	0.6368	ns	No	
Brain Region	19.23	0.0002	***	Yes	
ANOVA table	SS (Type III)	DF	MS	F (DFn, DFd)	P value
Interaction	1.868	5	0.3737	F (5, 101) = 2.583	P=0.0305
Drug Treatment	0.03245	1	0.03245	F (1, 101) = 0.2243	P=0.6368
Brain Region	3.953	5	0.7906	F (5, 101) = 5.466	P=0.0002
Residual	14.61	101	0.1446		

Šídák's multiple comparisons test	Predicted (LS) mean diff.	95.00% CI of diff.	DF	t	Adjusted P Value
Not Treated Rats: Region-specific effects					
Hippocampus vs. CA1	-0.2341	-0.7717 to 0.3035	101.0	1.306	0.9611
Hippocampus vs. CA3	0.3816	-0.1560 to 0.9192	101.0	2.129	0.4205
Hippocampus vs. DG	-0.1363	-0.6740 to 0.4013	101.0	0.7605	0.9999
Hippocampus vs. Subiculum	-0.1941	-0.7317 to 0.3435	101.0	1.083	0.9930
Hippocampus vs. DG Hilar Region	-0.3642	-0.9019 to 0.1734	101.0	2.032	0.4973
CA1 vs. CA3	0.6157	0.07812 to 1.153	101.0	3.434	0.0129
CA1 vs. DG	0.09777	-0.4398 to 0.6354	101.0	0.5453	>0.9999
CA1 vs. Subiculum	0.04000	-0.4976 to 0.5776	101.0	0.2231	>0.9999
CA1 vs. DG Hilar Region	-0.1301	-0.6677 to 0.4075	101.0	0.7258	>0.9999
CA3 vs. DG	-0.5180	-1.056 to 0.01965	101.0	2.889	0.0686
CA3 vs. Subiculum	-0.5757	-1.113 to -0.03812	101.0	3.211	0.0263
CA3 vs. DG Hilar Region	-0.7459	-1.283 to -0.2082	101.0	4.160	0.0010
DG vs. Subiculum	-0.05777	-0.5954 to 0.4798	101.0	0.3222	>0.9999
DG vs. DG Hilar Region	-0.2279	-0.7655 to 0.3097	101.0	1.271	0.9689
Subiculum vs. DG Hilar Region	-0.1701	-0.7077 to 0.3675	101.0	0.9489	0.9982
DZ/DIB Treated Rats: Region-specific effects					
Hippocampus vs. CA1	-0.5632	-1.073 to -0.05320	101.0	3.311	0.0191
Hippocampus vs. CA3	0.02021	-0.5038 to 0.5442	101.0	0.1157	>0.9999
Hippocampus vs. DG	0.002870	-0.5072 to 0.5129	101.0	0.01687	>0.9999
Hippocampus vs. Subiculum	-0.2755	-0.7855 to 0.2346	101.0	1.620	0.8213
Hippocampus vs. DG Hilar Region	0.01680	-0.4932 to 0.5268	101.0	0.09877	>0.9999
CA1 vs. CA3	0.5834	0.05943 to 1.107	101.0	3.339	0.0175
CA1 vs. DG	0.5661	0.05607 to 1.076	101.0	3.328	0.0181
CA1 vs. Subiculum	0.2878	-0.2223 to 0.7978	101.0	1.692	0.7715
CA1 vs. DG Hilar Region	0.5800	0.07000 to 1.090	101.0	3.410	0.0139
CA3 vs. DG	-0.01734	-0.5413 to 0.5067	101.0	0.09923	>0.9999
CA3 vs. Subiculum	-0.2957	-0.8197 to 0.2283	101.0	1.692	0.7715
CA3 vs. DG Hilar Region	-0.003412	-0.5274 to 0.5206	101.0	0.01953	>0.9999
DG vs. Subiculum	-0.2783	-0.7884 to 0.2317	101.0	1.636	0.8102
DG vs. DG Hilar Region	0.01393	-0.4961 to 0.5240	101.0	0.08189	>0.9999
Subiculum vs. DG Hilar Region	0.2923	-0.2178 to 0.8023	101.0	1.718	0.7522
Brain Region Analysis: Transgenic Not Treated vs. Transgenic DZ/DIB Treated					
HC: Not Treated vs. DZ/DIB Treated	0.008001	-0.3386 to 0.3546	101.0	0.04579	0.9636
CA1: Not Treated vs. DZ/DIB Treated	-0.3211	-0.6678 to 0.02553	101.0	1.838	0.0691
CA3: Not Treated vs. DZ/DIB Treated	-0.3534	-0.7091 to 0.002238	101.0	1.971	0.0514
DG: Not Treated vs. DZ/DIB Treated	0.1472	-0.1994 to 0.4939	101.0	0.8425	0.4015
SB: Not Treated vs. DZ/DIB Treated	-0.07334	-0.4200 to 0.2733	101.0	0.4197	0.6756
DG HL: Not Treated vs. DZ/DIB Treated	0.3890	0.04240 to 0.7357	101.0	2.226	0.0282

Supplemental Table 4 –Data was analyzed using a two-way ANOVA assessing hippocampal regions (HC, CA1, CA3, DG, HL, and SB) for changes in Amyloid beta (**Aβ**) levels in untreated and DZ/DB-treated transgenic rats. Sidak's multiple comparisons tests were used to assess post-hoc differences between treatment conditions and brain regions. Abbreviations: HC – hippocampus, CA – cornu ammonis, DG – Dentate gyrus, HL – DG hilar region, SB – subiculum. TGNT – transgenic not treated (11-months), n = 9; TGTR – transgenic DZ/DIB treated (11 months) n = 10.

Supplemental Table 5 – Tau neurofibrillary tangle (PHF1) percent positive signal across hippocampal regions at 11 months of age

Two-way ANVOA Analysis					
**Source of Variation**	**% of total variation**	**P value**	**P value summary**	**Significant?**	
Interaction	2.068	0.1512	ns	No	
drug treatment	0.4447	0.2262	ns	No	
brain region	70.35	<0.0001	****	Yes	
**ANOVA table**	**SS (Type III)**	**DF**	**MS**	**F (DFn, DFd)**	**P value**
Interaction	0.7719	4	0.1930	F (4, 89) = 1.726	P=0.1512
drug treatment	0.1660	1	0.1660	F (1, 89) = 1.485	P=0.2262
brain region	26.26	4	6.565	F (4, 89) = 58.73	P<0.0001
Residual	9.948	89	0.1118		

Šídák's multiple comparisons test	Predicted (LS) mean diff.	95.00% CI of diff.	DF	t	Adjusted P Value
Not Treated Rats: Region-specific effects					
Hippocampus vs. CA1	-1.391	-1.843 to -0.9381	89.00	8.823	<0.0001
Hippocampus vs. CA3	-1.389	-1.841 to -0.9363	89.00	8.812	<0.0001
Hippocampus vs. DG	-1.294	-1.747 to -0.8418	89.00	8.212	<0.0001
Hippocampus vs. Subiculum	-0.7698	-1.222 to -0.3173	89.00	4.884	<0.0001
CA1 vs. CA3	0.001833	-0.4506 to 0.4543	89.00	0.01163	>0.9999
CA1 vs. DG	0.09628	-0.3562 to 0.5487	89.00	0.6109	0.9996
CA1 vs. Subiculum	0.6208	0.1683 to 1.073	89.00	3.939	0.0016
CA3 vs. DG	0.09444	-0.3580 to 0.5469	89.00	0.5993	0.9997
CA3 vs. Subiculum	0.6189	0.1665 to 1.071	89.00	3.927	0.0017
DG vs. Subiculum	0.5245	0.07206 to 0.9769	89.00	3.328	0.0127
DZ/DIB Treated Rats: Region-specific effects					
Hippocampus vs. CA1	-1.517	-1.926 to -1.108	89.00	10.64	<0.0001
Hippocampus vs. CA3	-1.198	-1.607 to -0.7886	89.00	8.403	<0.0001
Hippocampus vs. DG	-1.051	-1.470 to -0.6312	89.00	7.192	<0.0001
Hippocampus vs. Subiculum	-0.9886	-1.398 to -0.5794	89.00	6.935	<0.0001
CA1 vs. CA3	0.3193	-0.08999 to 0.7285	89.00	2.240	0.2442
CA1 vs. DG	0.4666	0.04724 to 0.8859	89.00	3.194	0.0192
CA1 vs. Subiculum	0.5285	0.1193 to 0.9378	89.00	3.707	0.0036
CA3 vs. DG	0.1473	-0.2720 to 0.5667	89.00	1.009	0.9775
CA3 vs. Subiculum	0.2093	-0.2000 to 0.6185	89.00	1.468	0.7928
DG vs. Subiculum	0.06193	-0.3574 to 0.4813	89.00	0.4239	>0.9999
Brain Region Analysis: Transgenic Not Treated vs. Transgenic DZ/DIB Treated					
HC: Not Treated vs. DZ/DIB Treated	0.06443	-0.2342 to 0.3630	89.00	0.4288	0.6691
CA1: Not Treated vs. DZ/DIB Treated	-0.06215	-0.3607 to 0.2364	89.00	0.4136	0.6802
CA3: Not Treated vs. DZ/DIB Treated	0.2553	-0.04331 to 0.5539	89.00	1.699	0.0929
DG: Not Treated vs. DZ/DIB Treated	0.3082	0.002945 to 0.6134	89.00	2.006	0.0479
SB: Not Treated vs. DZ/DIB Treated	-0.1544	-0.4530 to 0.1442	89.00	1.028	0.3070

Supplemental Table 5 –Data was analyzed using a two-way ANOVA assessing hippocampal regions (HC, CA1, CA3, DG, and SB) to measure effects of DZ/DIB treatment on Tau (PHF1) levels. Sidak's multiple comparisons tests were used to assess post-hoc differences between treatment conditions and brain regions. Abbreviations: HC – hippocampus, CA – cornu ammonis, DG – Dentate gyrus, SB – subiculum. TGNT – transgenic not treated (11-months), n = 9; TGTR – transgenic DZ/DIB treated (11 months) n = 10.

Supplemental Table 6: DZ/DIB effects on NeuN signal across the hippocampus and subregions

Overall 4-way ANOVA Results						
Source	Type III Sum of Squares	df	Mean Square	F	Sig.	Partial Eta Squared
Corrected Model	690.209[a]	29	23.800	5.872	<.001	.414
Intercept	72046.067	1	72046.067	17774.412	<.001	.987
Genotype (WT vs. Transgenic)	141.474	1	141.474	34.903	<.001	.127
Age (4 months vs. 11 months)	.618	1	.618	.152	.697	.001
Treatment (untreated vs. DZ/DIB treated)	43.424	1	43.424	10.713	.001	.043
Region (HC, CA1, CA3, DG, SB)	337.702	4	84.425	20.829	<.001	.257
Genotype * Age * Treatment * Region	10.625	4	2.656	.655	.624	.011
Genotype * Age	21.688	1	21.688	5.351	.022	.022
Genotype * Treatment * Region	1.579	4	.395	.097	.983	.002
Genotype * Treatment	3.814	1	3.814	.941	.333	.004
Genotype * Region	17.465	4	4.366	1.077	.368	.018
Age * Region	21.231	4	5.308	1.309	.267	.021
Treatment * Region	11.310	4	2.828	.698	.594	.011
Error	976.859	241	4.053			
Total	81027.925	271				
Corrected Total	1667.069	270				

Pairwise Comparisons (Genotype x Age x Region x Treatment) – Sidak's Post-hoc tests									
								95% Confidence Interval for Difference	
Genotype	Age	Region	(I) Treatment	(J) Treatment	Mean Difference (I-J)	Std. Error	Sig.	Lower Bound	Upper Bound
Transgenic	11 Months	CA1	DZ/DIB	No treatment	-1.898	.905	.037	-3.680	-.115
		CA3	DZ/DIB	No treatment	-1.754	.905	.054	-3.536	.029
		Dentate Gyrus	DZ/DIB	No treatment	-.362	.905	.690	-2.144	1.421
		Hippocampus	DZ/DIB	No treatment	-.601	.935	.521	-2.444	1.242
		Subiculum	DZ/DIB	No treatment	-1.598	.905	.079	-3.381	.184
Wild-type	11 Months	CA1	DZ/DIB	No treatment	-1.307	.955	.172	-3.188	.574
		CA3	DZ/DIB	No treatment	-.849	.925	.360	-2.671	.973
		Dentate Gyrus	DZ/DIB	No treatment	-.039	.925	.967	-1.861	1.783
		Hippocampus	DZ/DIB	No treatment	-.761	.949	.423	-2.631	1.108
		Subiculum	DZ/DIB	No treatment	-.416	.949	.662	-2.285	1.454

Supplemental Table 6: Mature neuronal cell detection in the hippocampus with NeuN. A four-way ANOVA assessing DZ/DIB drug treatment, genotype, age, and brain region effects on neuronal loss (top table). Sidak's multiple comparisons tests were used for relevant post-hoc analyses (bottom table). Abbreviations: HC – hippocampus; CA – cornu ammonis; DG – dentate gyrus; SB – subiculum; TG – transgenic; WT – wild-type. DZ/DIB treated (Diazoxide/Dibenozylmethane); WTNT (4 months), n = 8; TGNT (4 months), n = 8; WTNT (11 months), n = 10; TGNT (11 months), n = 10; WTTR (11 months), n = 10; TGTR (11 months), n = 10.

Supplemental Table 7: Effects of DZ/DIB treatment and age on microglia morphology across the hippocampus and within its subregions

Tests of Between-Subjects Effects (Overall 5-way ANOVA)					
Source	Type III Sum of Squares	df	Mean Square	F	Sig.
Corrected Model	1.750E-5[a]	119	1.471E-7	214.750	<.001
Intercept	1.924E-5	1	1.924E-5	28098.237	<.001
Genotype (WT vs Transgenic)	6.981E-8	1	6.981E-8	101.936	<.001
Age (4 months vs. 11 Months)	3.692E-9	1	3.692E-9	5.391	.020
Treatment (Untreated vs. DZ/DIB)	.000	1	.000	.000	1.000
Region (HC, CA1, CA3, DG, SB)	3.318E-8	4	8.295E-9	12.112	<.001
Morphology (Ramified, Reactive, Amoeboid)	1.496E-5	3	4.985E-6	7279.614	<.001
Genotype * Age * Treatment * Region * Morphology	1.921E-8	15	1.281E-9	1.870	.023
Genotype * Age * Treatment * Region	.000	4	.000	.000	1.000
Genotype * Age	4.647E-8	1	4.647E-8	67.852	<.001
Genotype * Treatment * Region * Morphology	.000	15	.000	.000	1.000
Genotype * Treatment * Region	.000	4	.000	.000	1.000
Genotype * Treatment	1.960E-9	1	1.960E-9	2.861	.091
Genotype * Region * Morphology	.000	12	.000	.000	1.000
Genotype * Region	.000	4	.000	.000	1.000
Genotype * Morphology	4.281E-8	3	1.427E-8	20.836	<.001
Age * Region * Morphology	2.230E-8	15	1.487E-9	2.171	.006
Age * Region	.000	4	.000	.000	1.000
Treatment * Region	.000	4	.000	.000	1.000
Treatment * Region * Morphology	.000	12	.000	.000	1.000
Treatment * Morphology	.000	3	.000	.000	1.000
Region * Morphology	7.962E-8	12	6.635E-9	9.689	<.001
Error	6.554E-7	957	6.849E-10		
Total	3.980E-5	1077			
Corrected Total	1.816E-5	1076			

Pairwise Comparisons (Genotype vs. Age vs Region vs Morphology vs Treatment)									95% Confidence Interval for Difference	
Genotype	Age	Region	Morphology	(I) Treatment	(J) Treatment	Mean Difference (I-J)	Std. Error	Sig.	Lower Bound	Upper Bound
Transgenic	11 Months	CA1	All	DZ/DIB	No treatment	1.218E-6	.000	.919	-2.238E-5	2.481E-5
			Amoeboid	DZ/DIB	No treatment	-2.422E-6	.000	.840	-2.602E-5	2.117E-5
			Ramified	DZ/DIB	No treatment	6.653E-6	.000	.580	-1.694E-5	3.025E-5
			Reactive	DZ/DIB	No treatment	-3.014E-6	.000	.802	-2.661E-5	2.058E-5
		CA3	All	DZ/DIB	No treatment	-1.675E-5	.000	.164	-4.034E-5	6.849E-6
			Amoeboid	DZ/DIB	No treatment	-1.563E-6	.000	.899	-2.577E-5	2.265E-5
			Ramified	DZ/DIB	No treatment	-1.895E-5	.000	.115	-4.254E-5	4.652E-6
			Reactive	DZ/DIB	No treatment	-1.312E-6	.000	.915	-2.552E-5	2.290E-5
		Dentate Gyrus	All	DZ/DIB	No treatment	-1.204E-5	.000	.317	-3.564E-5	1.155E-5
			Amoeboid	DZ/DIB	No treatment	-1.186E-6	.000	.921	-2.478E-5	2.241E-5
			Ramified	DZ/DIB	No treatment	-2.959E-5	.000	.014	-5.319E-5	-5.992E-6
			Reactive	DZ/DIB	No treatment	-3.073E-6	.000	.798	-2.667E-5	2.052E-5
		Hippocampus	All	DZ/DIB	No treatment	-1.016E-5	.000	.399	-3.375E-5	1.344E-5
			Amoeboid	DZ/DIB	No treatment	3.940E-7	.000	.975	-2.382E-5	2.460E-5
			Ramified	DZ/DIB	No treatment	-1.099E-5	.000	.361	-3.459E-5	1.260E-5
			Reactive	DZ/DIB	No treatment	-8.892E-7	.000	.941	-2.449E-5	2.271E-5
		Subiculum	All	DZ/DIB	No treatment	-3.830E-6	.000	.750	-2.743E-5	1.977E-5
			Amoeboid	DZ/DIB	No treatment	6.085E-6	.000	.613	-1.751E-5	2.968E-5
			Ramified	DZ/DIB	No treatment	-1.700E-5	.000	.158	-4.060E-5	6.597E-6
			Reactive	DZ/DIB	No treatment	7.084E-6	.000	.556	-1.651E-5	3.068E-5
Wild-type	11 Months	CA1	All	DZ/DIB	No treatment	6.403E-6	.000	.594	-1.719E-5	3.000E-5
			Amoeboid	DZ/DIB	No treatment	2.390E-8	.000	.998	-2.357E-5	2.362E-5
			Ramified	DZ/DIB	No treatment	4.603E-6	.000	.702	-1.899E-5	2.820E-5

			Reactive	DZ/DIB	No treatment	1.775E-6	.000	.883	-2.182E-5	2.537E-5
		CA3	All	DZ/DIB	No treatment	2.435E-6	.000	.840	-2.116E-5	2.603E-5
			Amoeboid	DZ/DIB	No treatment	-8.537E-7	.000	.943	-2.445E-5	2.274E-5
			Ramified	DZ/DIB	No treatment	4.742E-6	.000	.693	-1.885E-5	2.834E-5
			Reactive	DZ/DIB	No treatment	-1.455E-6	.000	.904	-2.505E-5	2.214E-5
		Dentate Gyrus	All	DZ/DIB	No treatment	2.872E-6	.000	.811	-2.072E-5	2.647E-5
			Amoeboid	DZ/DIB	No treatment	1.051E-6	.000	.930	-2.255E-5	2.465E-5
			Ramified	DZ/DIB	No treatment	-6.131E-6	.000	.610	-2.973E-5	1.747E-5
			Reactive	DZ/DIB	No treatment	7.953E-6	.000	.508	-1.564E-5	3.155E-5
		Hippocampus	All	DZ/DIB	No treatment	5.392E-6	.000	.654	-1.820E-5	2.899E-5
			Amoeboid	DZ/DIB	No treatment	-3.776E-7	.000	.975	-2.397E-5	2.322E-5
			Ramified	DZ/DIB	No treatment	7.128E-7	.000	.953	-2.288E-5	2.431E-5
			Reactive	DZ/DIB	No treatment	5.053E-6	.000	.674	-1.854E-5	2.865E-5
		Subiculum	All	DZ/DIB	No treatment	-8.318E-6	.000	.489	-3.192E-5	1.528E-5
			Amoeboid	DZ/DIB	No treatment	-3.906E-7	.000	.974	-2.399E-5	2.321E-5
			Ramified	DZ/DIB	No treatment	-1.103E-5	.000	.359	-3.463E-5	1.257E-5
			Reactive	DZ/DIB	No treatment	3.102E-6	.000	.796	-2.049E-5	2.670E-5

Supplemental Table 7: A five-way ANOVA assessing DZ/DIB drug treatment, genotype, age, morphology, and brain region effects (top table). Sidak's multiple comparisons tests were used for relevant post-hoc analyses (bottom table). Abbreviations: HC – hippocampus; CA – cornu ammonis; DG – dentate gyrus; SB – subiculum; TG – transgenic; WT – wild-type. DZ/DIB treated (Diazoxide/Dibenozylmethane); WTNT – wild-type not treated; TGNT – transgenic not treated; WTTR – wild-type DZ/DIB treated; TGTR – transgenic DZ/DIB treated. WTNT (4 months), n = 8; TGNT (4 months), n = 8; WTNT (11 months), n = 10; TGNT (11 months), n = 10; WTTR (11 months), n = 10; TGTR (11 months), n = 10.

Supplemental Table 8: DZ/DIB treatment effects on full-length APP levels in 4- and 11-month rats

Three-way ANOVA analysis					
Source of Variation	% of total variation	P value	P value summary	Significant?	
age	7.436	0.0011	**	Yes	
treatment	5.137	0.0055	**	Yes	
genotype	58.37	<0.0001	****	Yes	
age x treatment	1.370	0.1374	ns	No	
age x genotype	0.1774	0.5882	ns	No	
treatment x genotype	0.004900	0.9282	ns	No	
age x treatment x genotype	2.419	0.0508	ns	No	
**ANOVA table**	**SS (Type III)**	**DF**	**MS**	**F (DFn, DFd)**	**P value**
age	1.425	1	1.425	F (1, 39) = 12.49	P=0.0011
treatment	0.9846	1	0.9846	F (1, 39) = 8.629	P=0.0055
genotype	11.19	1	11.19	F (1, 39) = 98.05	P<0.0001
age x treatment	0.2625	1	0.2625	F (1, 39) = 2.301	P=0.1374
age x genotype	0.03401	1	0.03401	F (1, 39) = 0.2981	P=0.5882
treatment x genotype	0.0009391	1	0.0009391	F (1, 39) = 0.008230	P=0.9282
age x treatment x genotype	0.4636	1	0.4636	F (1, 39) = 4.063	P=0.0508
Residual	4.450	39	0.1141		

Tukey's multiple comparisons test	Predicted (LS) mean diff.	95.00% CI of diff.	DF	q	Adjusted P Value
4 Months:Wild Type Not Treated vs. 4 Months:Transgenic Not Treated	-0.7157	-1.370 to -0.06102	39.00	4.949	0.0236
4 Months:Wild Type Not Treated vs. 4 Months:Wild-Type DZ/DIB Treated	-0.2318	-0.8560 to 0.3924	39.00	1.681	0.9304
4 Months:Wild Type Not Treated vs. 4 Months:Transgenic DZ/DIB Treated	-1.363	-1.988 to -0.7392	39.00	9.887	<0.0001
4 Months:Wild Type Not Treated vs. 11 Months:Wild Type Not Treated	-0.2457	-0.8700 to 0.3785	39.00	1.782	0.9078
4 Months:Wild Type Not Treated vs. 11 Months:Transgenic Not Treated	-1.467	-2.091 to -0.8430	39.00	10.64	<0.0001
4 Months:Wild Type Not Treated vs. 11 Months:Transgenic DZ/DIB Treated	-1.417	-2.042 to -0.7932	39.00	10.28	<0.0001
4 Months:Transgenic Not Treated vs. 4 Months:Transgenic DZ/DIB Treated	-0.6477	-1.302 to 0.007017	39.00	4.478	0.0543
4 Months:Transgenic Not Treated vs. 11 Months:Wild Type Not Treated	0.4700	-0.1847 to 1.125	39.00	3.249	0.3206
4 Months:Transgenic Not Treated vs. 11 Months:Transgenic Not Treated	-0.7515	-1.406 to -0.09682	39.00	5.196	0.0149
4 Months:Transgenic Not Treated vs. 11 Months:Wild-Type DZ/DIB Treated	0.1397	-0.5150 to 0.7944	39.00	0.9657	0.9970
4 Months:Transgenic Not Treated vs. 11 Months:Transgenic DZ/DIB Treated	-0.7017	-1.356 to -0.04705	39.00	4.852	0.0282
4 Months:Wild-Type DZ/DIB Treated vs. 4 Months:Transgenic DZ/DIB Treated	-1.132	-1.756 to -0.5074	39.00	8.206	<0.0001
4 Months:Wild-Type DZ/DIB Treated vs. 11 Months:Wild Type Not Treated	-0.01396	-0.6382 to 0.6103	39.00	0.1012	>0.9999
4 Months:Wild-Type DZ/DIB Treated vs. 11 Months:Transgenic Not Treated	-1.235	-1.860 to -0.6112	39.00	8.959	<0.0001
4 Months:Wild-Type DZ/DIB Treated vs. 11 Months:Wild-Type DZ/DIB Treated	-0.3442	-0.9685 to 0.2800	39.00	2.496	0.6461
4 Months:Wild-Type DZ/DIB Treated vs. 11 Months:Transgenic DZ/DIB Treated	-1.186	-1.810 to -0.5614	39.00	8.598	<0.0001
4 Months:Transgenic DZ/DIB Treated vs. 11 Months:Wild Type Not Treated	1.118	0.4934 to 1.742	39.00	8.105	<0.0001
4 Months:Transgenic DZ/DIB Treated vs. 11 Months:Transgenic Not Treated	-0.1038	-0.7281 to 0.5204	39.00	0.7530	0.9994
4 Months:Transgenic DZ/DIB Treated vs. 11 Months:Wild-Type DZ/DIB Treated	0.7873	0.1631 to 1.412	39.00	5.710	0.0055
4 Months:Transgenic DZ/DIB Treated vs. 11 Months:Transgenic DZ/DIB Treated	-0.05407	-0.6783 to 0.5702	39.00	0.3921	>0.9999
11 Months:Wild Type Not Treated vs. 11 Months:Transgenic Not Treated	-1.221	-1.846 to -0.5972	39.00	8.858	<0.0001
11 Months:Wild Type Not Treated vs. 11 Months:Wild-Type DZ/DIB Treated	-0.3303	-0.9545 to 0.2939	39.00	2.395	0.6911
11 Months:Wild Type Not Treated vs. 11 Months:Transgenic DZ/DIB Treated	-1.172	-1.796 to -0.5475	39.00	8.497	<0.0001
11 Months:Transgenic Not Treated vs. 11 Months:Wild-Type DZ/DIB Treated	0.8912	0.2670 to 1.515	39.00	6.463	0.0011
11 Months:Transgenic Not Treated vs. 11 Months:Transgenic DZ/DIB Treated	0.04977	-0.5745 to 0.6740	39.00	0.3609	>0.9999
11 Months:Wild-Type DZ/DIB Treated vs. 11 Months:Transgenic DZ/DIB Treated	-0.8414	-1.466 to -0.2172	39.00	6.102	0.0024

Supplemental Table 8: Tg-AD rats show higher levels of full-length APP at both 4-months and 11-months; DZ/DIB treatment attenuates increases in APP levels. Values represent the percentage of the pixel ratio for full-length APP over actin loading control. Data was assessed as a three-way ANOVA considering age (4 vs. 11 months), genotype (wild-type vs. transgenic), and drug treatment (no treatment vs. DZ/DIB treatment). Tukey's multiple comparisons test represent post-hoc analysis following the three-way ANOVA. *$p < 0.05$, **$p < 0.01$, ***$p < 0.001$, ****$p < 0.0001$. WTNT, wild-type not treated, TGNT, transgenic-AD not treated, WTTR, wild-type DZ/DIB treated, TGTR, transgenic-AD DZ/DIB treated.

Supplemental Table 9: DZ/DIB treatment effects on eIF2-alpha levels in 4- and 11-month rats

Source of Variation	% of total variation	P value	P value summary	Significant?	
age	3.686	0.0864	ns	No	
treatment	20.32	0.0002	***	Yes	
genotype	25.55	<0.0001	****	Yes	
age x treatment	0.4778	0.5302	ns	No	
age x genotype	0.3780	0.5764	ns	No	
treatment x genotype	0.09001	0.7848	ns	No	
age x treatment x genotype	2.027	0.1997	ns	No	
**ANOVA table**	**SS (Type III)**	**DF**	**MS**	**F (DFn, DFd)**	**P value**
age	0.3957	1	0.3957	F (1, 39) = 3.095	P=0.0864
treatment	2.181	1	2.181	F (1, 39) = 17.06	P=0.0002
genotype	2.742	1	2.742	F (1, 39) = 21.45	P<0.0001
age x treatment	0.05129	1	0.05129	F (1, 39) = 0.4012	P=0.5302
age x genotype	0.04057	1	0.04057	F (1, 39) = 0.3173	P=0.5764
treatment x genotype	0.009662	1	0.009662	F (1, 39) = 0.07557	P=0.7848
age x treatment x genotype	0.2176	1	0.2176	F (1, 39) = 1.702	P=0.1997
Residual	4.987	39	0.1279		

Tukey's multiple comparisons test	Predicted (LS) mean diff.	95.00% CI of diff.	DF	q	Adjusted P Value
4 Months:Wild Type Not Treated vs. 4 Months:Transgenic Not Treated	-0.3778	-1.039 to 0.2830	39.00	2.588	0.6044
4 Months:Wild Type Not Treated vs. 4 Months:Wild-Type DZ/DIB Treated	0.6629	0.002073 to 1.324	39.00	4.541	0.0488
4 Months:Wild Type Not Treated vs. 4 Months:Transgenic DZ/DIB Treated	-0.04505	-0.7059 to 0.6158	39.00	0.3086	>0.9999
4 Months:Wild Type Not Treated vs. 11 Months:Wild Type Not Treated	0.3275	-0.3656 to 1.021	39.00	2.139	0.7961
4 Months:Wild Type Not Treated vs. 11 Months:Transgenic Not Treated	-0.2052	-0.8660 to 0.4556	39.00	1.406	0.9726
4 Months:Wild Type Not Treated vs. 11 Months:Wild-Type DZ/DIB Treated	0.5853	-0.07548 to 1.246	39.00	4.010	0.1154
4 Months:Wild Type Not Treated vs. 11 Months:Transgenic DZ/DIB Treated	0.2678	-0.3930 to 0.9286	39.00	1.835	0.8944
4 Months:Transgenic Not Treated vs. 4 Months:Wild-Type DZ/DIB Treated	1.041	0.3799 to 1.702	39.00	7.129	0.0003
4 Months:Transgenic Not Treated vs. 4 Months:Transgenic DZ/DIB Treated	0.3328	-0.3281 to 0.9936	39.00	2.279	0.7405
4 Months:Transgenic Not Treated vs. 11 Months:Wild Type Not Treated	0.7053	0.01224 to 1.398	39.00	4.607	0.0436
4 Months:Transgenic Not Treated vs. 11 Months:Transgenic Not Treated	0.1726	-0.4882 to 0.8334	39.00	1.182	0.9898
4 Months:Transgenic Not Treated vs. 11 Months:Wild-Type DZ/DIB Treated	0.9631	0.3023 to 1.624	39.00	6.598	0.0009
4 Months:Transgenic Not Treated vs. 11 Months:Transgenic DZ/DIB Treated	0.6456	-0.01520 to 1.306	39.00	4.423	0.0596
4 Months:Wild-Type DZ/DIB Treated vs. 4 Months:Transgenic DZ/DIB Treated	-0.7079	-1.369 to -0.04712	39.00	4.849	0.0283
4 Months:Wild-Type DZ/DIB Treated vs. 11 Months:Wild Type Not Treated	-0.3354	-1.028 to 0.3577	39.00	2.191	0.7764
4 Months:Wild-Type DZ/DIB Treated vs. 11 Months:Transgenic Not Treated	-0.8681	-1.529 to -0.2073	39.00	5.947	0.0034
4 Months:Wild-Type DZ/DIB Treated vs. 11 Months:Wild-Type DZ/DIB Treated	-0.07755	-0.7384 to 0.5833	39.00	0.5312	>0.9999
4 Months:Wild-Type DZ/DIB Treated vs. 11 Months:Transgenic DZ/DIB Treated	-0.3951	-1.056 to 0.2657	39.00	2.706	0.5503
4 Months:Transgenic DZ/DIB Treated vs. 11 Months:Wild Type Not Treated	0.3726	-0.3205 to 1.066	39.00	2.433	0.6743
4 Months:Transgenic DZ/DIB Treated vs. 11 Months:Transgenic Not Treated	-0.1602	-0.8210 to 0.5006	39.00	1.097	0.9935
4 Months:Transgenic DZ/DIB Treated vs. 11 Months:Wild-Type DZ/DIB Treated	0.6304	-0.03043 to 1.291	39.00	4.318	0.0709
4 Months:Transgenic DZ/DIB Treated vs. 11 Months:Transgenic DZ/DIB Treated	0.3129	-0.3480 to 0.9737	39.00	2.143	0.7946
11 Months:Wild Type Not Treated vs. 11 Months:Transgenic Not Treated	-0.5327	-1.226 to 0.1603	39.00	3.480	0.2424
11 Months:Wild Type Not Treated vs. 11 Months:Wild-Type DZ/DIB Treated	0.2578	-0.4352 to 0.9509	39.00	1.684	0.9298
11 Months:Wild Type Not Treated vs. 11 Months:Transgenic DZ/DIB Treated	-0.05969	-0.7528 to 0.6334	39.00	0.3899	>0.9999
11 Months:Transgenic Not Treated vs. 11 Months:Wild-Type DZ/DIB Treated	0.7906	0.1297 to 1.451	39.00	5.416	0.0098
11 Months:Transgenic Not Treated vs. 11 Months:Transgenic DZ/DIB Treated	0.4730	-0.1878 to 1.134	39.00	3.240	0.3239
11 Months:Wild-Type DZ/DIB Treated vs. 11 Months:Transgenic DZ/DIB Treated	-0.3175	-0.9783 to 0.3433	39.00	2.175	0.7824

Supplemental Table 9: Tg-AD rats show higher levels of eIF2-alpha at both 4-months and 11-months; DZ/DIB treatment attenuates increases in eIF2-alpha. Values represent the percentage of the pixel ratio for eIF2-alpha over beta-tubulin loading control. Data was assessed as a three-way ANOVA considering age (4 vs. 11 months), genotype (wild-type vs. transgenic), and drug treatment (no treatment vs. DZ/DIB treatment). Tukey's multiple comparisons test represent post-hoc analysis following the three-way ANOVA. *$p < 0.05$, **$p < 0.01$, ***$p < 0.001$, ****$p < 0.0001$. WTNT, wild-type not treated, TGNT, transgenic-AD not treated, WTTR, wild-type DZ/DIB treated, TGTR, transgenic-AD DZ/DIB treated.

Supplemental Table 10A: Analysis of light working memory in 4-month and 11-month rats

Three-way ANOVA Analysis					
Source of Variation	% of total variation	P value	P value summary	Significant?	
age	1.530	0.1738	ns	No	
drug treatment	1.955	0.1248	ns	No	
genotype	11.79	0.0003	***	Yes	
age x drug treatment	4.598	0.0195	*	Yes	
age x genotype	2.531	0.0813	ns	No	
drug treatment x genotype	0.9011	0.2957	ns	No	
age x drug treatment x genotype	1.616	0.1623	ns	No	
**ANOVA table**	**SS (Type III)**	**DF**	**MS**	**F (DFn, DFd)**	**P value**
age	0.2433	1	0.2433	F (1, 96) = 1.877	P=0.1738
drug treatment	0.3107	1	0.3107	F (1, 96) = 2.398	P=0.1248
genotype	1.874	1	1.874	F (1, 96) = 14.46	P=0.0003
age x drug treatment	0.7309	1	0.7309	F (1, 96) = 5.640	P=0.0195
age x genotype	0.4023	1	0.4023	F (1, 96) = 3.104	P=0.0813
drug treatment x genotype	0.1432	1	0.1432	F (1, 96) = 1.105	P=0.2957
age x drug treatment x genotype	0.2569	1	0.2569	F (1, 96) = 1.983	P=0.1623
Residual	12.44	96	0.1296		

Tukey's multiple comparisons test	Predicted (LS) mean diff.	95.00% CI of diff.	DF	q	Adjusted P Value
4 Months:Wild-Type Not Treated vs. 4 Months:Transgenic Not Treated	-0.1233	-0.5065 to 0.2600	96.00	1.409	0.9739
4 Months:Wild-Type Not Treated vs. 4 Months:Wild-Type DZ/DIB Treated	-0.03438	-0.4841 to 0.4153	96.00	0.3350	>0.9999
4 Months:Wild-Type Not Treated vs. 4 Months:Transgenic DZ/DIB Treated	-0.2099	-0.5984 to 0.1787	96.00	2.367	0.7039
4 Months:Wild-Type Not Treated vs. 11 Months:Wild-Type Not Treated	0.1585	-0.2498 to 0.5667	96.00	1.701	0.9292
4 Months:Wild-Type Not Treated vs. 11 Months:Transgenic Not Treated	-0.4288	-0.8936 to 0.03598	96.00	4.043	0.0929
4 Months:Wild-Type Not Treated vs. 11 Months:Wild-Type DZ/DIB Treated	0.2656	-0.1992 to 0.7304	96.00	2.504	0.6410
4 Months:Wild-Type Not Treated vs. 11 Months:Transgenic DZ/DIB Treated	0.03835	-0.3986 to 0.4753	96.00	0.3847	>0.9999
4 Months:Transgenic Not Treated vs. 4 Months:Wild-Type DZ/DIB Treated	0.08889	-0.3511 to 0.5289	96.00	0.8854	0.9984
4 Months:Transgenic Not Treated vs. 4 Months:Transgenic DZ/DIB Treated	-0.08660	-0.4639 to 0.2907	96.00	1.006	0.9965
4 Months:Transgenic Not Treated vs. 11 Months:Wild-Type Not Treated	0.2817	-0.1158 to 0.6793	96.00	3.106	0.3633
4 Months:Transgenic Not Treated vs. 11 Months:Transgenic Not Treated	-0.3056	-0.7610 to 0.1499	96.00	2.940	0.4357
4 Months:Transgenic Not Treated vs. 11 Months:Wild-Type DZ/DIB Treated	0.3889	-0.06652 to 0.8443	96.00	3.742	0.1526
4 Months:Transgenic Not Treated vs. 11 Months:Transgenic DZ/DIB Treated	0.1616	-0.2653 to 0.5885	96.00	1.659	0.9376
4 Months:Wild-Type DZ/DIB Treated vs. 4 Months:Transgenic DZ/DIB Treated	-0.1755	-0.6201 to 0.2691	96.00	1.730	0.9230
4 Months:Wild-Type DZ/DIB Treated vs. 11 Months:Wild-Type Not Treated	0.1929	-0.2690 to 0.6547	96.00	1.830	0.8990
4 Months:Wild-Type DZ/DIB Treated vs. 11 Months:Transgenic Not Treated	-0.3944	-0.9070 to 0.1181	96.00	3.373	0.2608
4 Months:Wild-Type DZ/DIB Treated vs. 11 Months:Wild-Type DZ/DIB Treated	0.3000	-0.2125 to 0.8125	96.00	2.565	0.6124
4 Months:Wild-Type DZ/DIB Treated vs. 11 Months:Transgenic DZ/DIB Treated	0.07273	-0.4147 to 0.5601	96.00	0.6539	0.9998
4 Months:Transgenic DZ/DIB Treated vs. 11 Months:Wild-Type Not Treated	0.3683	-0.03425 to 0.7709	96.00	4.010	0.0984
4 Months:Transgenic DZ/DIB Treated vs. 11 Months:Transgenic Not Treated	-0.2190	-0.6788 to 0.2409	96.00	2.087	0.8185
4 Months:Transgenic DZ/DIB Treated vs. 11 Months:Wild-Type DZ/DIB Treated	0.4755	0.01564 to 0.9353	96.00	4.531	0.0374
4 Months:Transgenic DZ/DIB Treated vs. 11 Months:Transgenic DZ/DIB Treated	0.2482	-0.1834 to 0.6799	96.00	2.520	0.6337
11 Months:Wild-Type Not Treated vs. 11 Months:Transgenic Not Treated	-0.5873	-1.064 to -0.1107	96.00	5.400	0.0056
11 Months:Wild-Type Not Treated vs. 11 Months:Wild-Type DZ/DIB Treated	0.1071	-0.3695 to 0.5837	96.00	0.9852	0.9969
11 Months:Wild-Type Not Treated vs. 11 Months:Transgenic DZ/DIB Treated	-0.1201	-0.5696 to 0.3293	96.00	1.171	0.9911
11 Months:Transgenic Not Treated vs. 11 Months:Wild-Type DZ/DIB Treated	0.6944	0.1686 to 1.220	96.00	5.787	0.0022
11 Months:Transgenic Not Treated vs. 11 Months:Transgenic DZ/DIB Treated	0.4672	-0.03422 to 0.9686	96.00	4.083	0.0866
11 Months:Wild-Type DZ/DIB Treated vs. 11 Months:Transgenic DZ/DIB Treated	-0.2273	-0.7287 to 0.2741	96.00	1.986	0.8530

Supplemental Table 10B: Analysis of heavy working memory in 4-month and 11-month rats

Source of Variation	% of total variation	P value	P value summary	Significant?	
Treatment	0.02295	0.8648	ns	No	
Age	15.17	<0.0001	****	Yes	
Genotype	5.443	0.0099	**	Yes	
Treatment x Age	1.785	0.1353	ns	No	
Treatment x Genotype	0.001987	0.9600	ns	No	
Age x Genotype	1.151	0.2294	ns	No	
Treatment x Age x Genotype	0.02898	0.8482	ns	No	
**ANOVA table**	**SS (Type III)**	**DF**	**MS**	**F (DFn, DFd)**	**P value**
Treatment	0.07600	1	0.07600	F (1, 97) = 0.02916	P=0.8648
Age	50.24	1	50.24	F (1, 97) = 19.28	P<0.0001
Genotype	18.02	1	18.02	F (1, 97) = 6.916	P=0.0099
Treatment x Age	5.912	1	5.912	F (1, 97) = 2.269	P=0.1353
Treatment x Genotype	0.006579	1	0.006579	F (1, 97) = 0.002525	P=0.9600
Age x Genotype	3.813	1	3.813	F (1, 97) = 1.463	P=0.2294
Treatment x Age x Genotype	0.09594	1	0.09594	F (1, 97) = 0.03682	P=0.8482
Residual	252.8	97	2.606		

Tukey's multiple comparisons test	Predicted (LS) mean diff.	95.00% CI of diff.	DF	q	Adjusted P Value
Untreated:11 month WT vs. Untreated:11 month TG	1.210	-0.9265 to 3.347	97.00	2.482	0.6516
Untreated:11 month WT vs. Untreated:4 month WT	1.277	-0.5277 to 3.082	97.00	3.101	0.3655
Untreated:11 month WT vs. Untreated:4 month TG	1.821	-0.2493 to 3.892	97.00	3.854	0.1276
Untreated:11 month WT vs. DZ/DIB Treated:11 month WT	-0.5952	-2.732 to 1.542	97.00	1.221	0.9886
Untreated:11 month WT vs. DZ/DIB Treated:11 month TG	0.7078	-1.307 to 2.723	97.00	1.539	0.9578
Untreated:11 month WT vs. DZ/DIB Treated:4 month WT	1.794	0.01145 to 3.576	97.00	4.410	0.0474
Untreated:11 month WT vs. DZ/DIB Treated:4 month TG	2.179	0.3743 to 3.984	97.00	5.290	0.0072
Untreated:11 month TG vs. Untreated:4 month WT	0.06699	-1.995 to 2.129	97.00	0.1424	>0.9999
Untreated:11 month TG vs. Untreated:4 month TG	0.6111	-1.687 to 2.909	97.00	1.165	0.9913
Untreated:11 month TG vs. DZ/DIB Treated:11 month WT	-1.806	-4.163 to 0.5521	97.00	3.355	0.2666
Untreated:11 month TG vs. DZ/DIB Treated:11 month TG	-0.5025	-2.750 to 1.745	97.00	0.9795	0.9970
Untreated:11 month TG vs. DZ/DIB Treated:4 month WT	0.5833	-1.458 to 2.625	97.00	1.252	0.9868
Untreated:11 month TG vs. DZ/DIB Treated:4 month TG	0.9690	-1.093 to 3.031	97.00	2.059	0.8283
Untreated:4 month WT vs. Untreated:4 month TG	0.5441	-1.449 to 2.537	97.00	1.196	0.9899
Untreated:4 month WT vs. DZ/DIB Treated:11 month WT	-1.873	-3.934 to 0.1891	97.00	3.979	0.1035
Untreated:4 month WT vs. DZ/DIB Treated:11 month TG	-0.5695	-2.505 to 1.366	97.00	1.289	0.9843
Untreated:4 month WT vs. DZ/DIB Treated:4 month WT	0.5163	-1.175 to 2.208	97.00	1.337	0.9806
Untreated:4 month WT vs. DZ/DIB Treated:4 month TG	0.9020	-0.8135 to 2.617	97.00	2.304	0.7317
Untreated:4 month TG vs. DZ/DIB Treated:11 month WT	-2.417	-4.715 to -0.1187	97.00	4.608	0.0320
Untreated:4 month TG vs. DZ/DIB Treated:11 month TG	-1.114	-3.299 to 1.072	97.00	2.233	0.7616
Untreated:4 month TG vs. DZ/DIB Treated:4 month WT	-0.02778	-2.000 to 1.945	97.00	0.06170	>0.9999
Untreated:4 month TG vs. DZ/DIB Treated:4 month TG	0.3578	-1.635 to 2.351	97.00	0.7866	0.9993
DZ/DIB Treated:11 month WT vs. DZ/DIB Treated:11 month TG	1.303	-0.9449 to 3.551	97.00	2.540	0.6244
DZ/DIB Treated:11 month WT vs. DZ/DIB Treated:4 month WT	2.389	0.3471 to 4.431	97.00	5.126	0.0105
DZ/DIB Treated:11 month WT vs. DZ/DIB Treated:4 month TG	2.775	0.7128 to 4.836	97.00	5.896	0.0017
DZ/DIB Treated:11 month TG vs. DZ/DIB Treated:4 month WT	1.086	-0.8282 to 3.000	97.00	2.486	0.6497
DZ/DIB Treated:11 month TG vs. DZ/DIB Treated:4 month TG	1.471	-0.4638 to 3.407	97.00	3.331	0.2752
DZ/DIB Treated:4 month WT vs. DZ/DIB Treated:4 month TG	0.3856	-1.306 to 2.077	97.00	0.9989	0.9966

Supplemental Table 10: DZ/DIB treatment mitigates spatial working memory deficits at 11 months of age. The number of working memory errors under a light working memory load (A) and heavy working memory load (B) were tabulated for each experimental group (WTNT, TGNT, WTTR, and TGTR) at 4 months and 11-months. 3-way ANOVA analysis is used assess effects of age, drug treatment, and genotype in RAM performance under both the light- and heavy-working memory loads. Tukey's multiple comparisons tests were used to find post-hoc differences. *$p < 0.05$, **$p < 0.01$, ***$p < 0.001$, ****$p < 0.0001$. WTNT, wild-type not treated, TGNT, transgenic-AD not treated, WTTR, wild-type DZ/DIB treated, TGTR, transgenic-AD DZ/DIB treated.

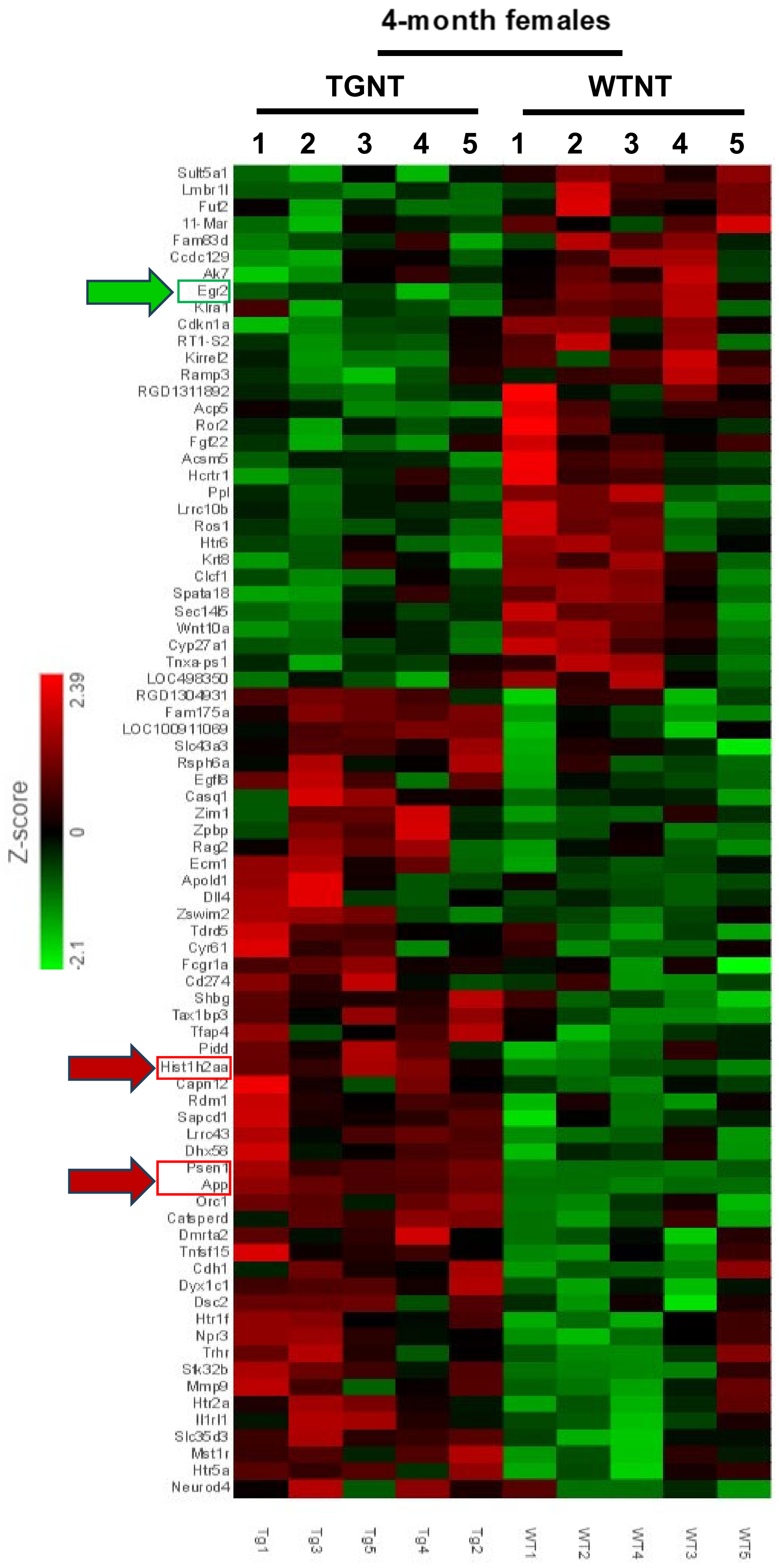

Supplemental Figure 1: Heatmap based on Z-scores for differentially expressed genes in 4-month female Tg rats when compared to wild type. Based on the Z-scores, the downregulated genes are in green and the upregulated genes are in red. The green arrow indicates a significant downregulation for Egr2, and the red arrows indicate a significant upregulation for Hist1h2aa, APP and Psen1.

A

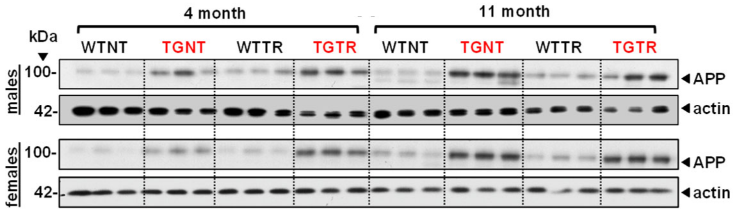

B

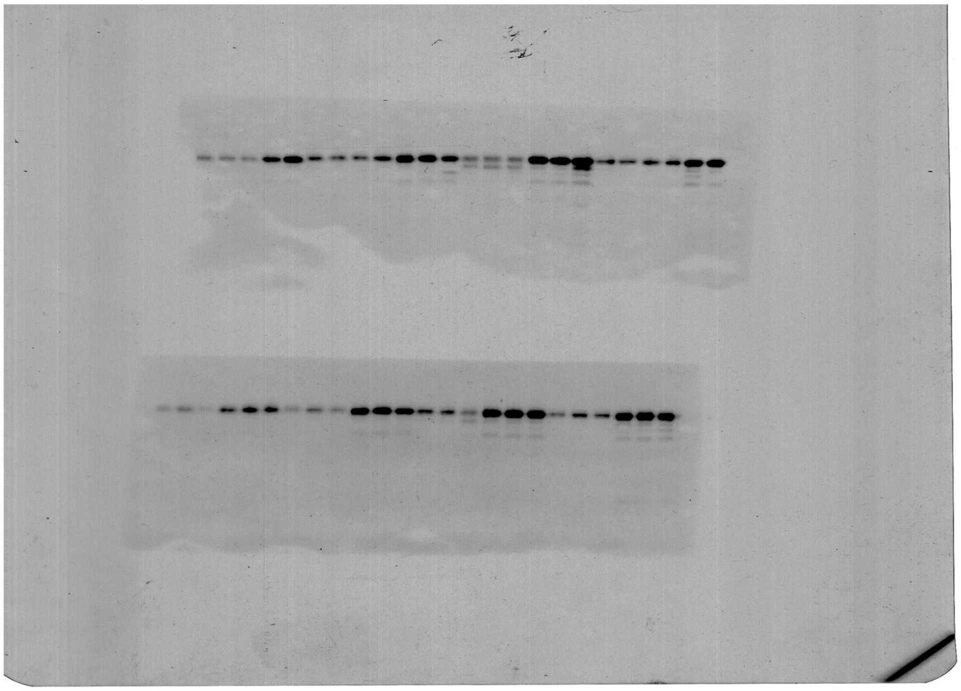

Supplemental Figure 2: A – Panel shown in Figure 5A of the manuscript. **B** - Scan of the film used to visualize full length-APP on the two western blots placed in the same film developing cassette. The dark exposure shows the edges of the blots. The line on the bottom right corner was used to mark the film's orientation. Hippocampal tissue was analyzed by western blotting. Male (top gel) and female (bottom gel) rats were included. Abbreviations: APP – amyloid precursor protein; WTNT – wild-type not treated, TGNT – transgenic not treated, WTTR – wild-type DZ/DIB treated, TGTR – transgenic DZ/DIB treated.

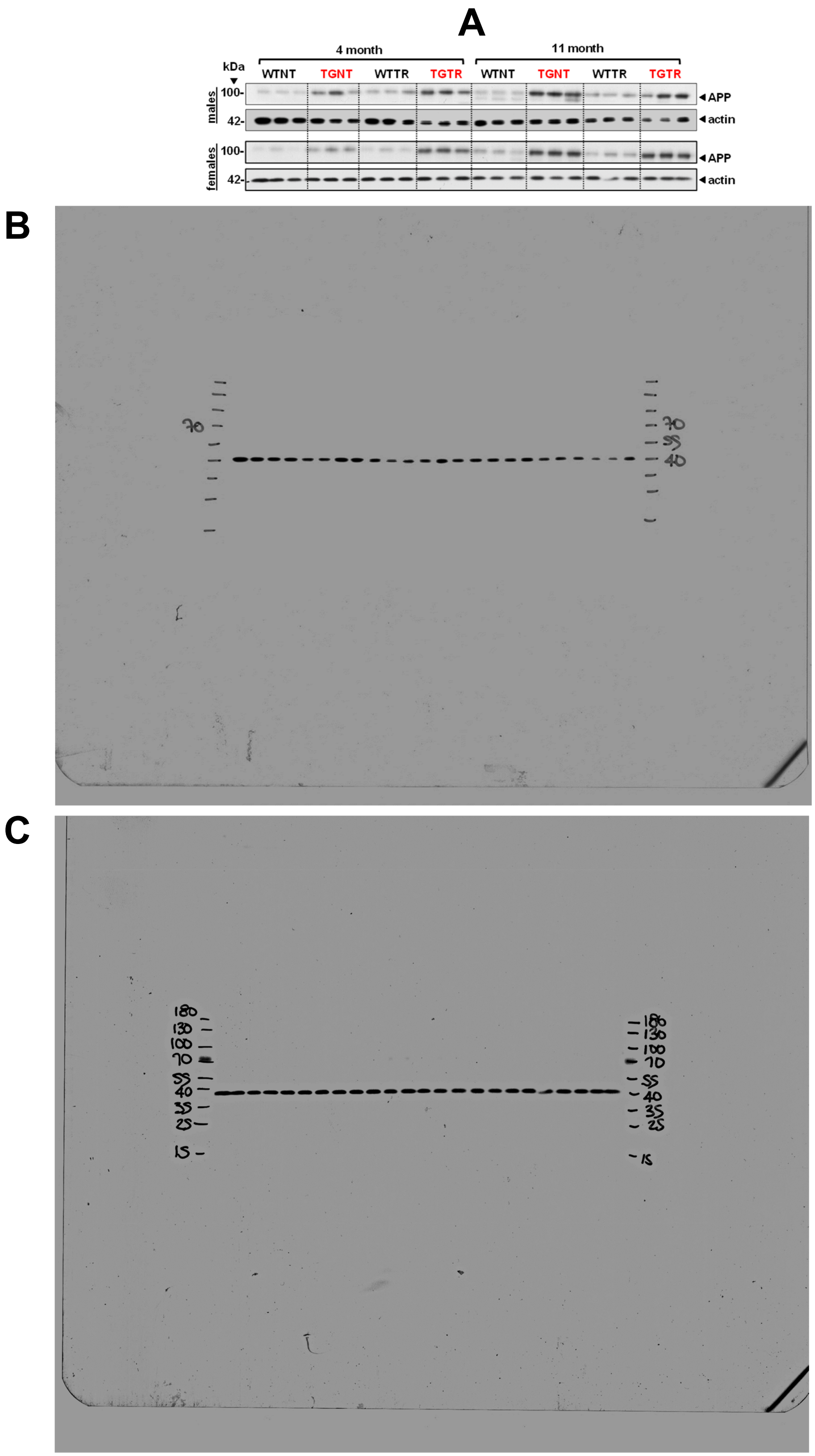

Supplemental Figure 3: A – Panel shown in Figure 5A of the manuscript. **B and C** - Scans of the films used to visualize actin on the two western blots. The dark exposure shows the edges of the films. Molecular weight markers are labelled on the left and right of the blots. The line on the bottom right corner was used to mark the film's orientation. Hippocampal tissue was analyzed by western blotting. Male (top gel) and female (bottom gel) rats were included. Abbreviations: APP – amyloid precursor protein; WTNT – wild-type not treated, TGNT – transgenic not treated, WTTR – wild-type DZ/DIB treated, TGTR – transgenic DZ/DIB treated.

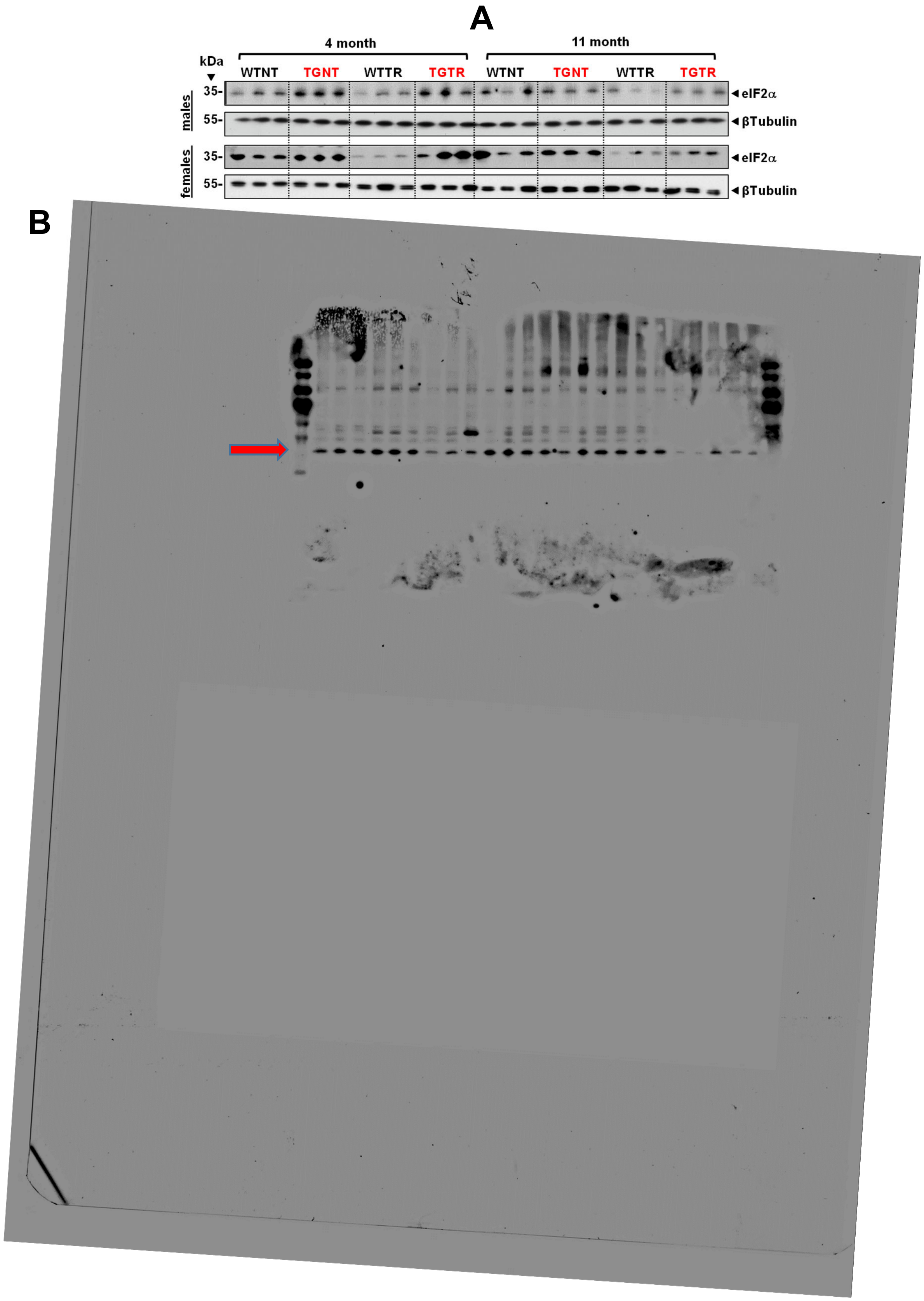

Supplemental Figure 4: A – Panel shown in Figure 6A of the manuscript. **B** - Scan of the film used to visualize eIF2α on the male western blot. The dark exposure shows the edges of the film. The line on the bottom left corner was used to mark the film's orientation. Hippocampal tissue was analyzed by western blotting. Abbreviations: eIF2α - eukaryotic initiation factor 2α, WTNT – wild-type not treated, TGNT – transgenic not treated, WTTR – wild-type DZ/DIB treated, TGTR – transgenic DZ/DIB treated. Red arrow, eIF2α based on its molecular weight.

A

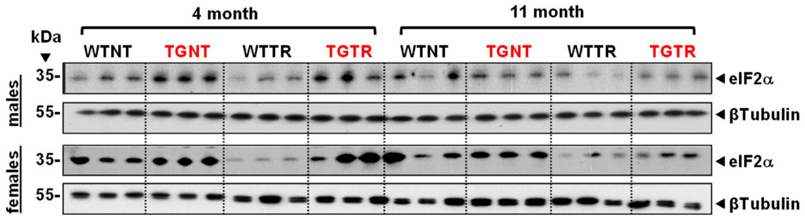

B

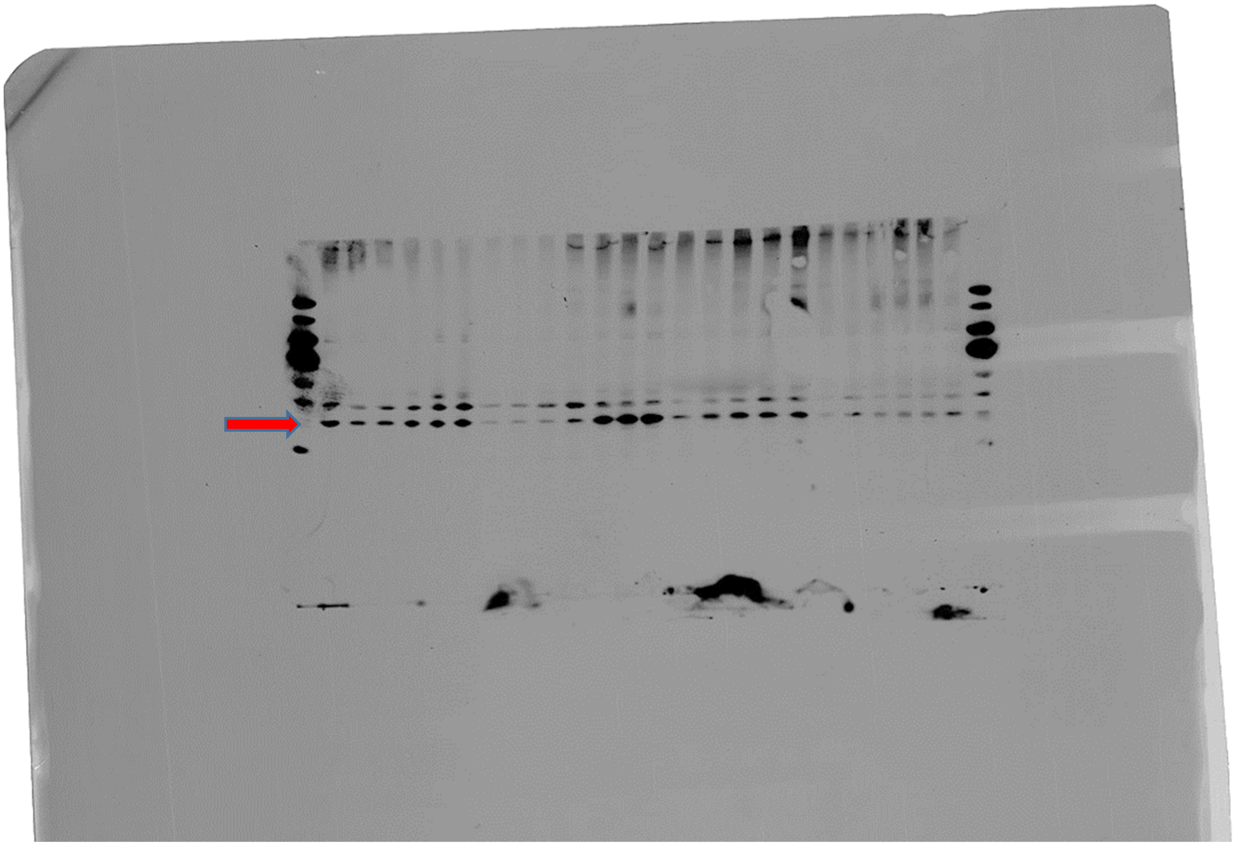

Supplemental Figure 5: A – Panel shown in Figure 6A of the manuscript. **B** - Scan of the film used to visualize eIF2α on the female western blot. The dark exposure shows the edges of the film. The line on the top left corner was used to mark the film's orientation. Hippocampal tissue was analyzed by western blotting. Abbreviations: eIF2α - eukaryotic initiation factor 2α, WTNT – wild-type not treated, TGNT – transgenic not treated, WTTR – wild-type DZ/DIB treated, TGTR – transgenic DZ/DIB treated. Red arrow, eIF2α based on its molecular weight.

A

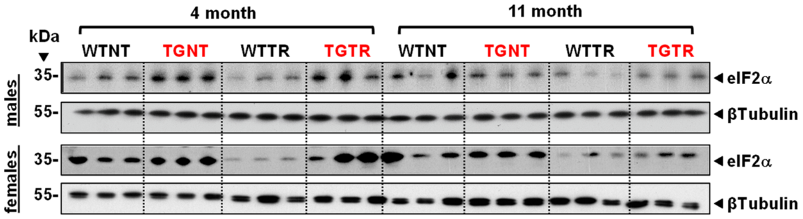

B

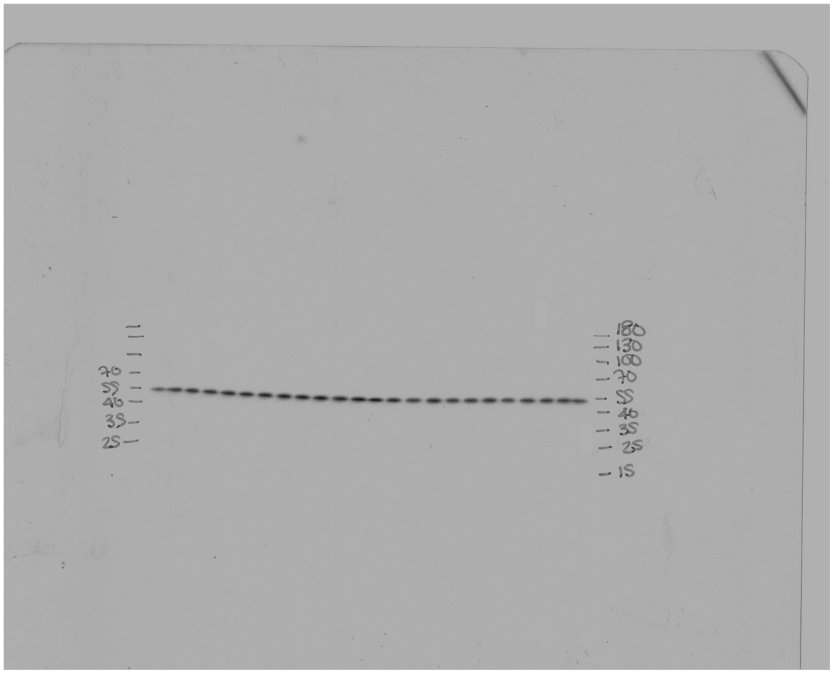

C

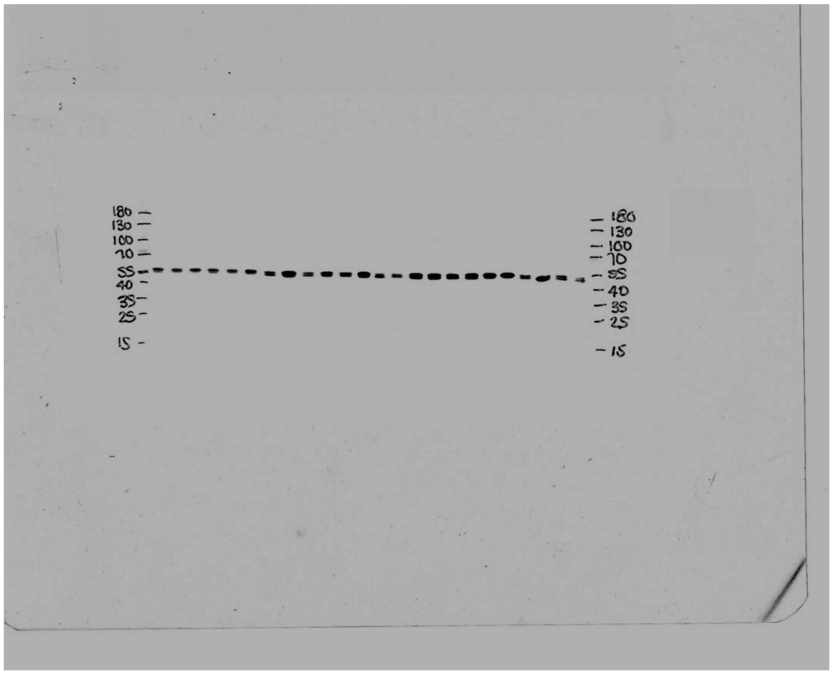

Supplemental Figure 6: A – Panel shown in Figure 6A of the manuscript. **B and C** - Scans of the films used to visualize βtubulin on the two western blots. The dark exposure shows the edges of the films. Molecular weight markers are labelled on the left and right of the blots. The diagonal lines on the corners were used to mark the film's orientation. Hippocampal tissue was analyzed by western blotting. Male (top gel) and female (bottom gel) rats were included. Abbreviations: eIF2α - eukaryotic initiation factor 2α; WTNT – wild-type not treated, TGNT – transgenic not treated, WTTR – wild-type DZ/DIB treated, TGTR – transgenic DZ/DIB treated.

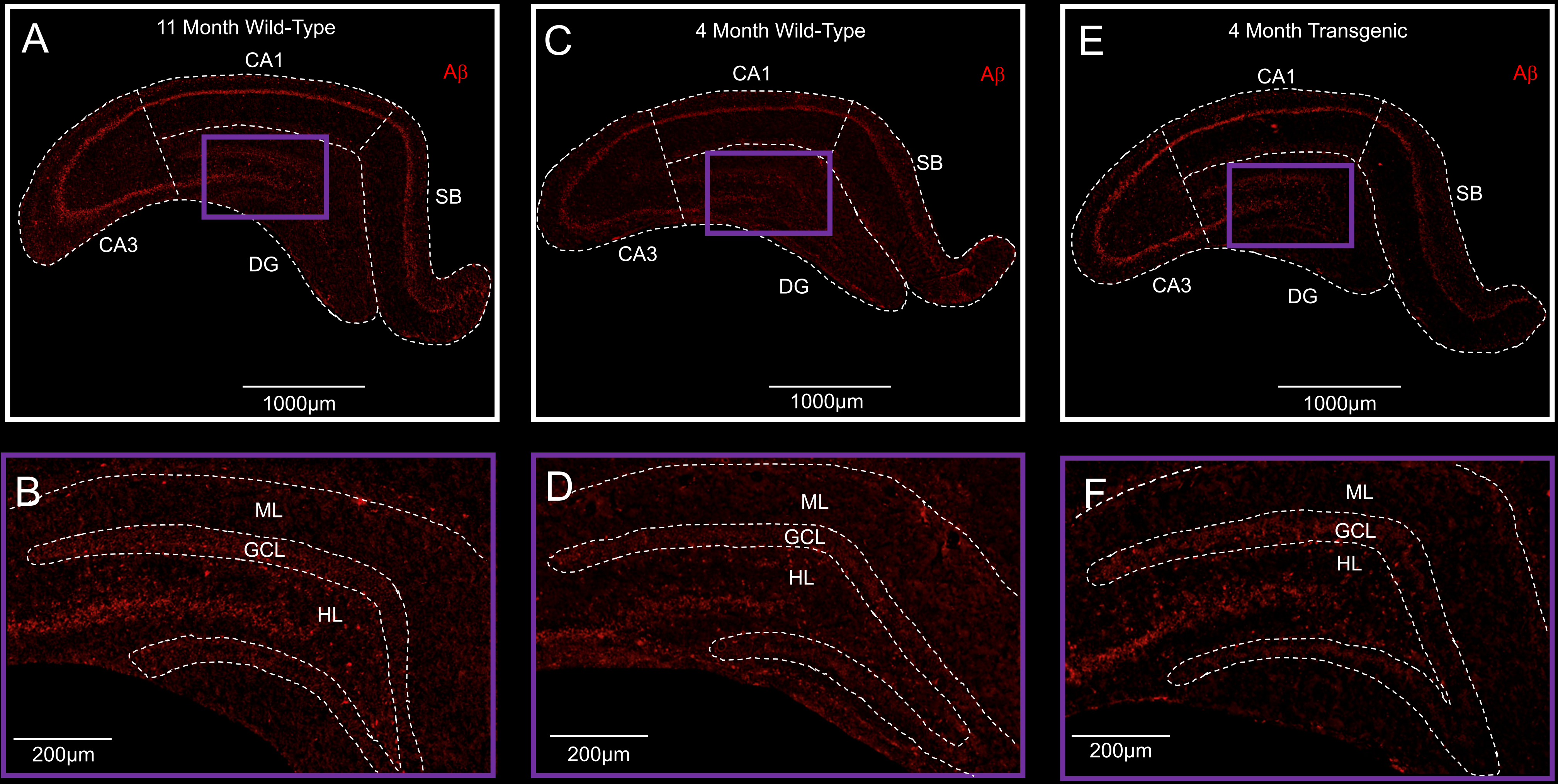

Supplemental Figure 7: 11 month-old wild-type (A and B), 4-month Wild-type (C and D), and 4-month transgenic (E and F) rats stained by IHC for Amyloid Beta across hippocampal regions CA1, CA3, DG, and SB. Scale bar = 1000μm (panels A, C, and E). Scale bar = 200μm (panels B, D and F). Full hippocampus are visible in panels A, C and E, with magnifications of the DG (panels B, D, and F) below the respective hippocampal image. CA = cornu ammonis, DG = dentate gyrus, SB = subiculum.

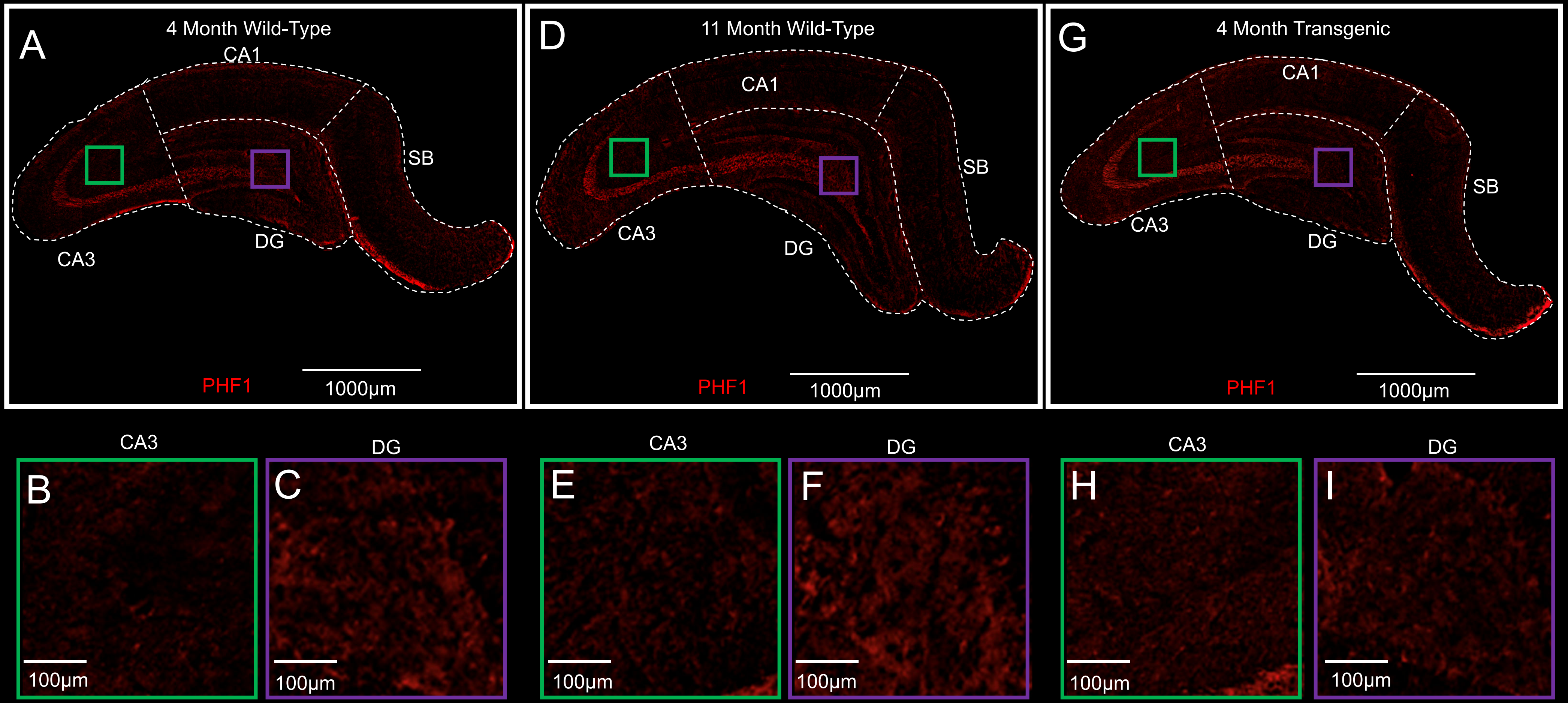

Supplemental Figure 8: 4 month-old wild-type (A, B, and C), 11-month Wild-type (D, E, and F), and 4-month transgenic (G, H, and I) rats stained by IHC for Tau paired helical filaments (PHF1), an early precursor of neurofibrillary tangles across hippocampal regions CA1, CA3, DG, and SB. Scale bar = 1000µm (panels A, D and G). Scale bar = 100µm (panels B, C, E, F, H, and I). Full hippocampus are visible in panels A, D and G, with magnifications of CA3 (panels B, E, and H) and the DG (panels C, F, and I) below the respective hippocampal image. CA = cornu ammonis, DG = dentate gyrus, SB = subiculum.
